# A Decline in HIV and Syphilis Epidemics in Chinese Female Sex Workers (2000–2011): A Systematic Review and Meta-Analysis

**DOI:** 10.1371/journal.pone.0082451

**Published:** 2013-12-13

**Authors:** Zongxing Yang, Junwei Su, Xiaorong Peng, Nanping Wu

**Affiliations:** State Key Laboratory for Diagnosis and Treatment of Infectious Diseases, the First Affiliated Hospital, School of Medicine, Zhejiang University, Hangzhou, Zhejiang Province, China; University of Southern California Keck School of Medicine, United States of America

## Abstract

**Background:**

Female sex workers (FSWs) play an important role in transmitting HIV and syphilis from high-risk groups to the general population. However, the trends in HIV and syphilis epidemics in Chinese FSWs in the period after 2000 are unclear to date.

**Methods:**

The Preferred Reporting Items for Systematic Reviews and Meta-Analyses Statement was followed. Seven databases were searched for published peer-reviewed articles. The incidence of HIV and syphilis in FSWs in different time periods, provinces and workplaces in China were separately pooled by meta-analysis. Correlation analysis was conducted between HIV and syphilis incidence and study time, respectively.

**Results:**

After 1,662 articles were screened, 190 published papers were included in the final analysis. Estimated HIV prevalence was 0.284% (95% CI: 0.080–0.488%) in the period 2000–2002, 0.211% (95% CI: 0.149–0.273%) in 2003–2005, 0.242% (95% CI: 0.190–0.294%) in 2006–2008 and 0.041% (95% CI: 0.024–0.058%) in 2009–2011. The corresponding syphilis prevalence was 9.669% (95% CI: 7.810–11.529%), 4.970% (95% CI: 4.384–5.556%), 4.404% (95% CI: 4.032–4.775%) and 3.169% (95% CI: 2.738–3.600%), respectively. Spearman rank correlation coefficients were −0.165 (*p* = 0.002) between HIV prevalence and study time, and −0.209 (*p* = 0.000) between syphilis prevalence and study time. The combined HIV prevalence was 0.318% (95% CI: 0.156–0.479%) in medium and high-tier workplaces and 0.393% (95% CI: 0.176–0.610%) in low-tier workplaces. The corresponding syphilis prevalence was 3.216% (95% CI: 2.192–4.240%) and 13.817% (95% CI: 10.589–17.044%), respectively.

**Conclusions:**

Our data suggested a decline in HIV and syphilis epidemics in FSWs in China on a national level during the study period (2000–2011). FSWs in low-tier workplaces should be given more attention in the future to ensure they are included in prevention programs for HIV and sexually transmitted diseases.

## Introduction

The Joint United Nations Programme on HIV/AIDS (UNAIDS) estimated that in 33 countries HIV incidence had declined by more than 25% in the past decade, and epidemiological surveillance showed a decline in the rate of spread of HIV between 2002 and 2010 [1], [2]. In China, since the first AIDS case was reported in 1985, HIV epidemics have gone through four phases: the sporadic phase (1985–1988), the localized epidemic phase (1989–1993), the spreading phase (1994–2000), and the fourth phase (2001–) [3]. At the end of 2011, the estimated number of people living with HIV/AIDS in China (PLHIV) was 0.78 million. They remained concentrated in several high-risk populations: commercial sex workers, injecting drug users (IDUs), former plasma donors (FPDs) and men who have sex with men [4]. Another important sexually transmitted disease (STD) in China – syphilis, can greatly increase the efficiency of HIV transmission [5]. China's national surveillance, in which active surveillance and passive surveillance are combined, showed that the incidence of syphilis was 32.04/100,000 per year in 2011, an increase of 10.89% compared to 2010, and it was among the first three Chinese notifiable diseases [6].

In China, female sex workers (FSWs) play an important bridging role in the transmission of HIV/STD from high-risk groups to the general population, given the importance of heterosexual transmission as the primary driver of HIV epidemics in China since 2007 and the reemergence and flourishing of commercial sex following the reform and opening-up policy [4], [7]. Meta-analysis suggested a high-risk of HIV infection (OR = 50) in Chinese FSWs compared to women of reproductive age or those aged 15–49 years [8]. However, the chronological trends in HIV and syphilis prevalence in Chinese FSWs since 2000 have not been investigated at a national level. The main objective of our study was to explore the epidemiological trends in HIV and syphilis infection in FSWs in China in the period from 2000 to 2011.

## Materials and Methods

We conducted and reported this systematic review according to the Preferred Reporting Items for Systematic Reviews and Meta-Analyses (PRISMA) Statement [9] (Table S1).

### Search strategy

Seven databases, including the Cochrane Library, PubMed, Chinese Biomedical Literature Service System (SinoMed), Wanfang Data, Chinese Scientific Journals Fulltext Database (CQVIP), China National Knowledge Infrastructure (CNKI) and Web of Knowledge, were searched by two investigators (Z.Y. and X.P.) for published peer-reviewed articles. The following terms were used in our search: (“commercial sex worker” OR “female sex worker” OR “sex work*”) AND (“HIV” OR “AIDS”) AND (“sexually transmitted diseases” OR “STD” OR “sexually transmitted infection” OR “syphilis”) AND “China”. We have submitted the detailed search strategy for PubMed in Table S2. In addition, the reference lists of published articles were scanned. The most recent search was carried out on August 2, 2012.

### Study selection and quality assessment

Titles and abstracts of all the searched articles were first screened, and then the full texts were screened by two independent reviewers (Z.Y. and J.S.). Disagreements between reviewers were resolved by discussion.

Articles were included if they met the following eligibility criteria: (1) HIV and syphilis prevalence among FSWs in mainland China (geopolitical area under the jurisdiction of the People's Republic of China but not including Hong Kong, Taiwan and Macau) were both reported; (2) the diagnosis of HIV and syphilis infection was based on positive serological tests and, for syphilis, results of either nontreponemal tests or treponemal tests were all included in our study; (3) study design, sample size, study period and study location were all reported; (4) peer-reviewed journal articles; (5) articles published in Chinese or English; (6) studies conducted between 2000 and 2011.

We excluded case reports, studies conducted before 2000 or after 2011, reviews, PhD or Master's theses, conference abstracts, studies not conducted in mainland China, studies with repeated data and reports that were not peer-reviewed. Additionally, studies with diagnosis based on oral fluid, urine or other non-serological tests were excluded. If the same data was published in both Chinese and English, we excluded the Chinese articles.

We considered studies to be of high quality according to the following criteria: (1) studies using probability sampling, especially those reporting detailed sampling methods; (2) studies with a sample size greater than 200; (3) the exact serological testing methods for the diagnosis of HIV and syphilis infection were reported; (4) two or more testing methods (including confirmatory tests) were used to diagnose HIV or syphilis infection; (5) cross-sectional studies without repetition or cohort studies; (6) studies with a loss rate of less than 10% or studies with a response rate of more than 90%. The closer the above criteria were adhered to, the higher the quality of the study.

### Data extraction

Two authors (Z.Y. and X.P.) independently extracted the data. Disagreements were resolved by discussion between them; if no agreement could be reached, a third author (N.W.) made the decision.

We extracted the following information from each study included: (1) first author, year of publication, language of publication, study period and province(s) where the study was conducted; (2) study design, study location (entertainment venues, reeducation centers or others) and sampling methods; (3) sample size, and the corresponding HIV and syphilis prevalence; (4) workplaces, and the corresponding sample size and HIV and syphilis prevalence; (5) testing methods for HIV and syphilis infection; (6) for the intervention studies and cohort studies, in particular, we only extracted the baseline data.

We categorized the studies by geographical location into seven groups (North China, Northeast, East China, Central China, South China, Southwest and Northwest) according to the provinces where the studies were conducted. We also categorized the studies into four three-year periods: 2000–2002, 2003–2005, 2006–2008 and 2009–2011, according to the periods when the study was carried out. We categorized testing methods for syphilis and HIV infection into three groups each. For syphilis infection, these were: treponemal tests (*Treponema pallidum* particle agglutination assay – TPPA, enzyme-linked immunosorbent assay – ELISA, *Treponema pallidum* hemagglutination assay – TPHA, immunocolloidal gold); nontreponemal tests (rapid plasma reagin – RPR, toluidine red unheated serum test – TRUST, unheated serum reagin – USR); and unspecified (exact methods not presented although diagnosis of syphilis infection was based on positive serological tests). For HIV infection the three groups were: confirmatory tests (ELISA + Western blot, ELISA-1 + ELISA-2, ELISA-1 + ELISA-2 + Western blot, immunocolloidal gold + Western blot, ELISA + immunocolloidal gold + Western blot); single method (single ELISA); and unspecified (exact methods not presented although diagnosis of HIV infection was based on positive serological tests).

In addition, we divided the studies into two groups based on the FSW's workplaces: medium and high-tier (with FSWs working in establishments such as hotels, Karaoke halls, salons, leisure centers, massage parlors, night clubs, dancing halls, bars, bath centers and footbath rooms); and low-tier (with FSWs working in the streets, rental houses, inns, lanes- narrow streets or alleys with poor housing conditions and a low house rent, lakefronts and rural entertainment venues).

### Statistical analysis

The estimates of HIV and syphilis prevalence and 95% CI were calculated using the random effect models (Q test *p*<0.10) or with the fixed effect models (Q test *p*>0.10). The Q test and *I* ^2^ statistic were used to test heterogeneity between studies. Subgroup analysis and meta-regression were performed to investigate potential sources of heterogeneity for all studies included. Spearman rank correlation coefficients (*r_s_*) were calculated to assess the relationship between HIV prevalence and syphilis prevalence, HIV prevalence and study time, and syphilis prevalence and study time, respectively. STATA (version 12) was used to perform meta-analysis, and a standard correction of 0.5 was added to all zero cells. Spearman rank correlation coefficients were calculated with SPSS (version 18.0). The significance levels were 0.10 for the Q test and 0.05 for the other tests.

## Results

### Flow of included studies

**Figure 1:**
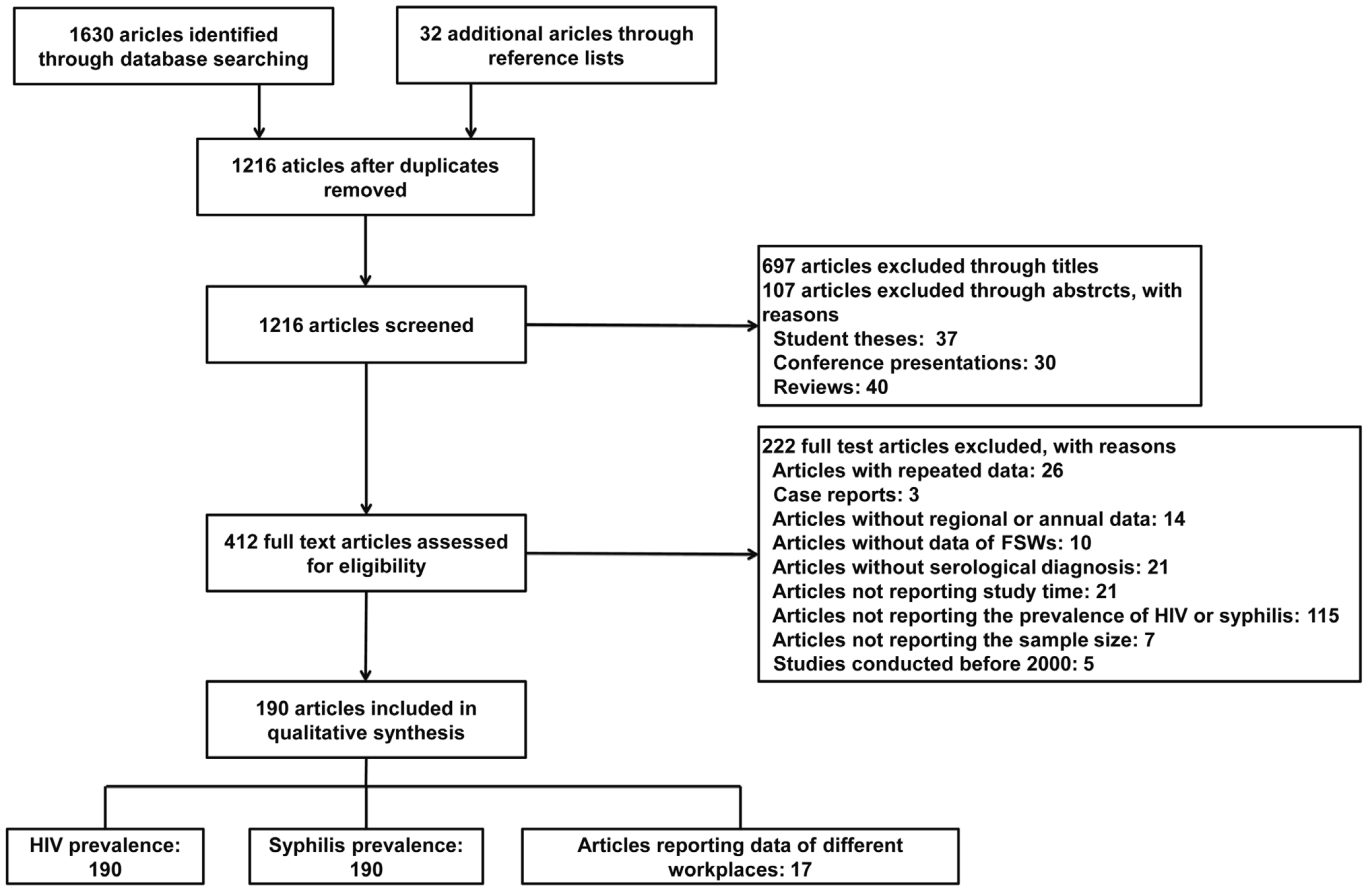
Diagram showing the selection of studies for meta-analysis.

As illustrated in Figure 1, we identified 1,630 articles from seven electronic databases using the search strategy, and 32 additional articles from the reference lists of identified articles. Once 446 duplicated articles had been discarded, the remaining 1,216 article titles and abstracts were screened. In this screening process 804 articles were excluded, as 697 articles clearly did not meet the criteria from the titles and 107 articles were judged to be ineligible by their abstracts. Four hundred and twelve articles were included in the full text screening and, of these, 222 articles were excluded with reasons illustrated in Figure 1. Finally, 190 articles were included in the quantitative synthesis; of these, 17 articles reported data from different workplaces and one of the 17 articles reported only HIV prevalence in different workplaces, although data on syphilis was collected simultaneously.

### Study characteristics

The 190 articles included covered all 31 provinces in mainland China except two (Tibet and Qinghai Province). Among the 190 articles included, 181 were cross-sectional studies and the other nine comprised eight intervention studies and one cohort study. In total, 341 records were collected (Table S3). Study periods covered every year from 2000 to 2011. Publication dates were between 2002 and 2012, and 179 articles were published in Chinese and 11 in English. Sample size ranged from 49 to 6,705 (median 363). One hundred and fifty studies were conducted in entertainment venues, 26 in reeducation centers and 14 in other locations. Fifty-one studies were performed with multistage sampling, four with respondent-driven sampling (RDS) or snowball sampling and 135 with convenience sampling.

In addition, 17 out of 190 articles reported the workplaces of the FSWs; these were published between 2005 and 2012. From the 17 articles, 40 records of HIV prevalence (medium and high-tier, 21, and low-tier, 19) and 37 records of syphilis prevalence (medium and high-tier, 19, and low-tier, 18) were collected (Table S4).

### Epidemic trends and regional discrepancies

**Figure 2:**
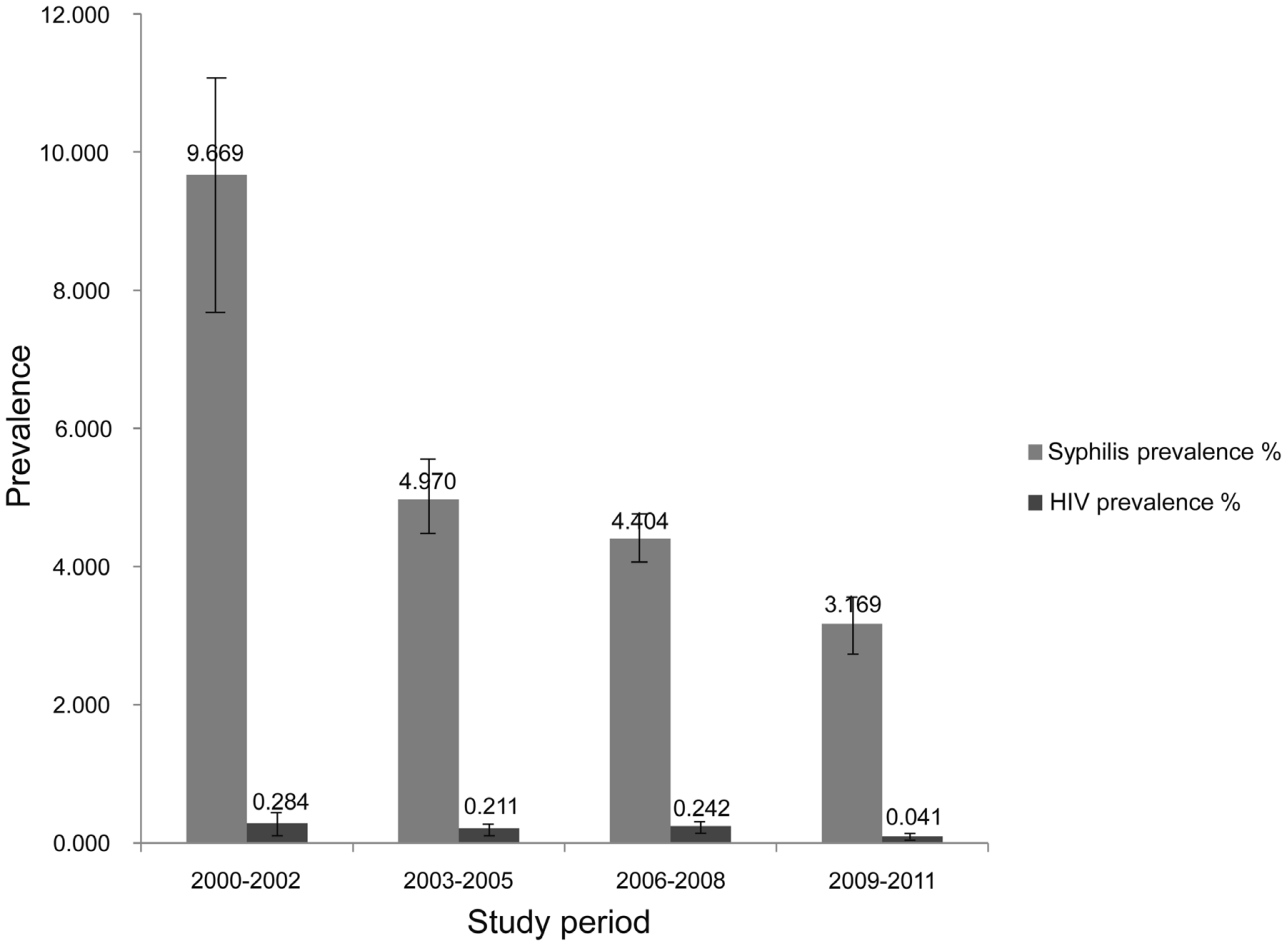
Estimated HIV and syphilis prevalence among Chinese female sex workers over the study period. The study period was divided into four three-year periods: 2000–2002, 2003–2005, 2006–2008 and 2009–2011. Each column represents the pooled estimate from meta-analysis of all studies over the corresponding time period. The error bars represent 95% confidence intervals of the percentages.

HIV prevalence was 0.284% (95% CI: 0.080–0.488%) in the period 2000–2002, 0.211% (95% CI: 0.149–0.273%) in 2003–2005, 0.242% (95% CI: 0.190–0.294%) in 2006–2008 and 0.041% (95% CI: 0.024–0.058%) in 2009–2011 (Figure 2). The overall estimated HIV prevalence was 0.203% (95% CI: 0.172–0.233%). *r_s_* between HIV prevalence and study time was −0.165 (*p* = 0.002, 2-tailed). The highest HIV prevalence was observed in Yunnan province (4.793%, 95% CI: 3.348–6.238%), and the other six provinces with an HIV prevalence above 0.2% were Chongqing (0.982%, 95% CI: 0.036–1.928%), Guangxi (0.450%, 95% CI: 0.311–0.590%), Sichuan (0.426%, 95%CI: 0.196–0.657%), Xinjiang (0.358%, 95% CI: 0.179–0.537%), Zhejiang (0.218%, 95% CI: 0.101–0.336%) and Hainan (0.215%, 95% CI: 0.058–0.372%), as can be seen in Table 1.

**Table 1:** Estimated prevalence of syphilis and HIV infection in female sex workers in 29 provinces in mainland China based on data from 2000 to 2011.

Province (No. of datasets)	Syphilis prevalence % (95% CI) *	Province (No. of datasets)	HIV prevalence % (95% CI)*
Hainan (14)	18.754 (13.409, 24.100)	Yunnan (21)	4.793 (3.348, 6.238)
Guizhou (2)	10.872 (6.419, 15.234)	Chongqing (4)	0.982 (0.036, 1.928)
Shanghai (12)	10.612 (7.252, 13.937)	Guangxi (30)	0.450 (0.311, 0.590)
Fujian (9)	10.359 (5.423, 15.295)	Sichuan (12)	0.426 (0.196, 0.657)
Zhejiang (19)	9.143 (6.734, 11.552)	Xinjiang (15)	0.358 (0.179, 0.537)
Gansu (1)	9.000 (7.381, 10.619)	Zhejiang (19)	0.218 (0.101, 0.336)
Anhui (21)	7.772 (6.137, 9.371)	Hainan (14)	0.215 (0.058, 0.372)
Inner Mongolia (3)	6.438 (3.518, 9.359)	Guizhou† (2)	0.204 (−0.138, 0.547)
Tianjin (13)	6.349 (5.474, 7.224)	Hebei† (1)	0.200 (−0.354, 0.754)
Guangdong (32)	5.204 (4.190, 6.218)	Henan† (5)	0.194 (−0.016, 0.405)
Sichuan (12)	5.051 (3.674, 6.428)	Guangdong (32)	0.183 (0.115, 0.251)
Guangxi (30)	4.778 (3.796, 5.760)	Fujian (9)	0.169 (0.011, 0.327)
Yunnan (21)	4.751 (3.318, 6.183)	Ningxia† (6)	0.154 (−0.019, 0.326)
Jiangsu (20)	4.589 (3.204, 5.974)	Shanghai (12)	0.150 (0.028, 0.273)
Hebei (1)	3.200 (1.018, 5.382)	Shanxi (17)	0.141 (0.044, 0.238)
Hubei (12)	3.192 (2.373, 4.010)	Jilin† (6)	0.137 (−0.021, 0.296)
Xinjiang (15)	3.130 (2.607, 4.193)	Tianjin (13)	0.134 (0.036, 0.231)
Beijing (20)	2.782 (2.092, 3.472)	Jiangsu (20)	0.129 (0.054, 0.205)
Chongqing (4)	2.464 (0.629, 4.299)	Shaanxi† (3)	0.126 (−0.075, 0.327)
Heilongjiang (5)	2.436 (1.548, 3.324)	Liaoning† (7)	0.120 (−0.013, 0.253)
Hunan (8)	2.170 (1.024, 3.316)	Heilongjiang† (5)	0.115 (−0.280, 0.258)
Shandong (18)	1.859 (1.399, 2.319)	Hubei (12)	0.113 (0.019, 0.207)
Shaanxi† (3)	1.519 (−0.099, 3.138)	Inner Mongolia† (3)	0.109 (−0.059, 0.276)
Ningxia (6)	1.451 (0.329, 2.573)	Hunan† (8)	0.106 (−0.003, 0.245)
Henan (5)	1.223 (0.698, 1.748)	Anhui (21)	0.044 (0.007, 0.082)
Shanxi (17)	1.036 (0.621, 1.452)	Jiangxi (5)	0.043 (0.003, 0.084)
Jiangxi (5)	1.010 (0.362, 1.657)	Gansu† (1)	0.042 (−0.074, 0.157)
Jilin (6)	0.668 (0.321, 1.016)	Beijing (20)	0.036 (0.005, 0.068)
Liaoning (7)	0.504 (0.043, 0.964)	Shandong† (18)	0.015 (−0.001, 0.030)
Overall (341)	4.475 (4.125, 4.735)	Overall (341)	0.203 (0.172, 0.233)

*Prevalence in descending order.

† *p*>0.05.

Syphilis prevalence decreased from 9.669% (95% CI: 7.81–11.529%) in the period 2000–2002 to 4.970% (95% CI: 4.384–5.556%) in 2003–2005, to 4.404% (95% CI: 4.032–4.775%) in 2006–2008, and to 3.169% (95% CI: 2.738–3.600%) in 2009–2011 (Figure 2), with an overall combined prevalence of 4.475% (95% CI: 4.125–4.735%). *r_s_* was −0.209 (*p* = 0.000, 2-tailed) between syphilis prevalence and study time. The highest combined syphilis prevalence was in Hainan (18.754%, 95% CI: 13.409–24.100%). Syphilis prevalence above 10% was observed in three provinces: Guizhou (10.872%, 95% CI: 6.419–15.234%), Shanghai (10.612%, 95% CI: 7.252–13.937%) and Fujian (10.359%, 95% CI: 5.423–15.295%), as can be seen in Table 1.

In addition, *r_s_* between HIV prevalence and syphilis prevalence was 0.263 (*p* = 0.000, 2-tailed).

### Heterogeneity between studies, subgroup analysis, and meta-regression analysis

For datasets reporting HIV prevalence, heterogeneity (Q test *p* = 0.000, *I* ^2^ = 65.000%) were observed. Meta-regression suggested that the language of publication (*p*-value from meta-regression of English: 0.002), sample size (*p*-value from meta-regression of studies with a sample size ≥400: 0.016) and geographical location (*p*-value from meta-regression of South and Southwest China: 0.001 and 0.000, respectively) might be the sources of heterogeneity (Table S5).

For datasets reporting syphilis prevalence, heterogeneity (Q test *p* = 0.000, *I* ^2^ = 94.100%) were also observed. Meta-regression suggested that study location (*p*-value from meta-regression of reeducation centers: 0.000) and geographical location (*p*-value from meta-regression of North China: 0.011) might have caused the heterogeneity (Table S5).

Subgroup analysis showed discrepancies between subgroups for both HIV prevalence and syphilis prevalence (Table S5). To control the confounders between subgroups we pooled HIV and syphilis prevalence by subgroups among the four three-year periods (Table S6) and calculated the Spearman rank correlation coefficients (*r_s_*) between HIV/syphilis prevalence and study time (2000–2011) for each subgroup (Table 2). As Table 2 shows, all the *r_s_* which were statistically significant suggested a downward trend in HIV or syphilis epidemics over the study periods in the corresponding subgroups. Furthermore, we analyzed the distribution of the 341 datasets and total sample sizes in the subgroups (Language, Sample size, Study location, Geographical location, Study design, Sampling methods, HIV testing methods and Syphilis testing methods) and the four three-year periods, to observe whether an unbalanced distribution of datasets and sample sizes in different subgroups between the four study periods contributed to the downward trend in HIV and syphilis epidemics (Table S7). As Table S7 shows, there were more datasets and greater sample sizes in the 2006–2008 and 2009–2011 study periods.

**Table 2:** Spearman rank correlation coefficients (*r_s_*) between HIV/syphilis prevalence and study time (2000–2011) in each subgroup.

	HIV		Syphilis	
Subgroup	*r_s_*	*p*-value	*r_s_*	*p*-value
**Language**				
Chinese	−0.137	**0.013**	−0.194	**0.000**
English	−0.747	**0.003**	−0.896	**0.000**
**Sample size**				
<200	0.165	0.197	−0.160	0.209
200–400	−0.133	0.086	−0.153	**0.048**
≥400	−0.139	0.148	−0.242	**0.011**
**Study location**				
Entertainment Venues	−0.144	**0.020**	0.035	0.570
Reeducation Centers	0.190	0.124	0.075	0.548
Others*	−0.489	0.064	−0.652	**0.008**
**Geographical location**				
East China	−0.264	**0.007**	−0.235	**0.016**
South China	0.015	0.898	−0.422	**0.000**
Southwest	−0.314	0.052	−0.472	**0.002**
Central China	−0.114	0.558	0.009	0.968
Northeast	−0.645	**0.004**	0.324	0.190
Northwest	−0.553	**0.004**	−0.297	0.149
North China	0.333	0.811	−0.116	0.403
**Study design**				
Cross-sectional studies	−0.176	**0.001**	−0.210	**0.000**
Others†	0.094	0.810	−0.034	0.930
**Sampling methods**				
Multistage sampling	−0.222	0.094	−0.205	0.122
Convenience sampling	−0.121	**0.043**	−0.195	**0.001**
RDS/snowball sampling‡	−0.900	**0.037**	−0.900	**0.037**
**HIV testing methods** §				
Confirmatory test	−0.172	**0.004**	-	-
Single method	−0.266	0.208	-	-
Unspecified	−0.182	0.274	-	-
**Syphilis testing methods** ||				
Treponemal tests	-	-	−0.396	**0.000**
Nontreponemal tests	-	-	−0.024	0.770
Unspecified	-	-	−0.263	0.176
**Overall**	−0.165	**0.002**	−0.209	**0.000**

*Comprising “Unspecified (6), Entertainment Venues and Reeducation Centers (6), Medical Institutions (1), Reeducation Centers and STD Clinics (1) and STD Clinics (1)”.

† Comprising “Cohort Study (1) and Intervention Study (8)”.

‡ RDS: Respondent Driven Sampling.

§ Sorted into three groups: Confirmatory tests (ELISA + Western blot, ELISA-1 + ELISA-2, ELISA-1 + ELISA-2 + Western blot, Immunocolloidal gold + Western blot, ELISA + Immunocolloidal gold + Western blot); Single method (single ELISA); and Unspecified (exact methods not presented although diagnosis of HIV infection based on positive serological tests).

|| Sorted into three groups: Treponemal tests (*Treponema pallidum* particle agglutination assay – TPPA, Enzyme-Linked Immuno Sorbent Assay – ELISA, *Treponema pallidum* hemagglutination assay – TPHA, Immunocolloidal gold); Nontreponemal tests (Rapid plasma reagin – RPR, Toluidine red unheated serum test – TRUST, Unheated serum reagin – USR); and Unspecified (exact methods not presented although diagnosis of syphilis infection based on positive serological tests).

### Estimated prevalence of HIV and syphilis infection in different workplaces

**Figure 3:**
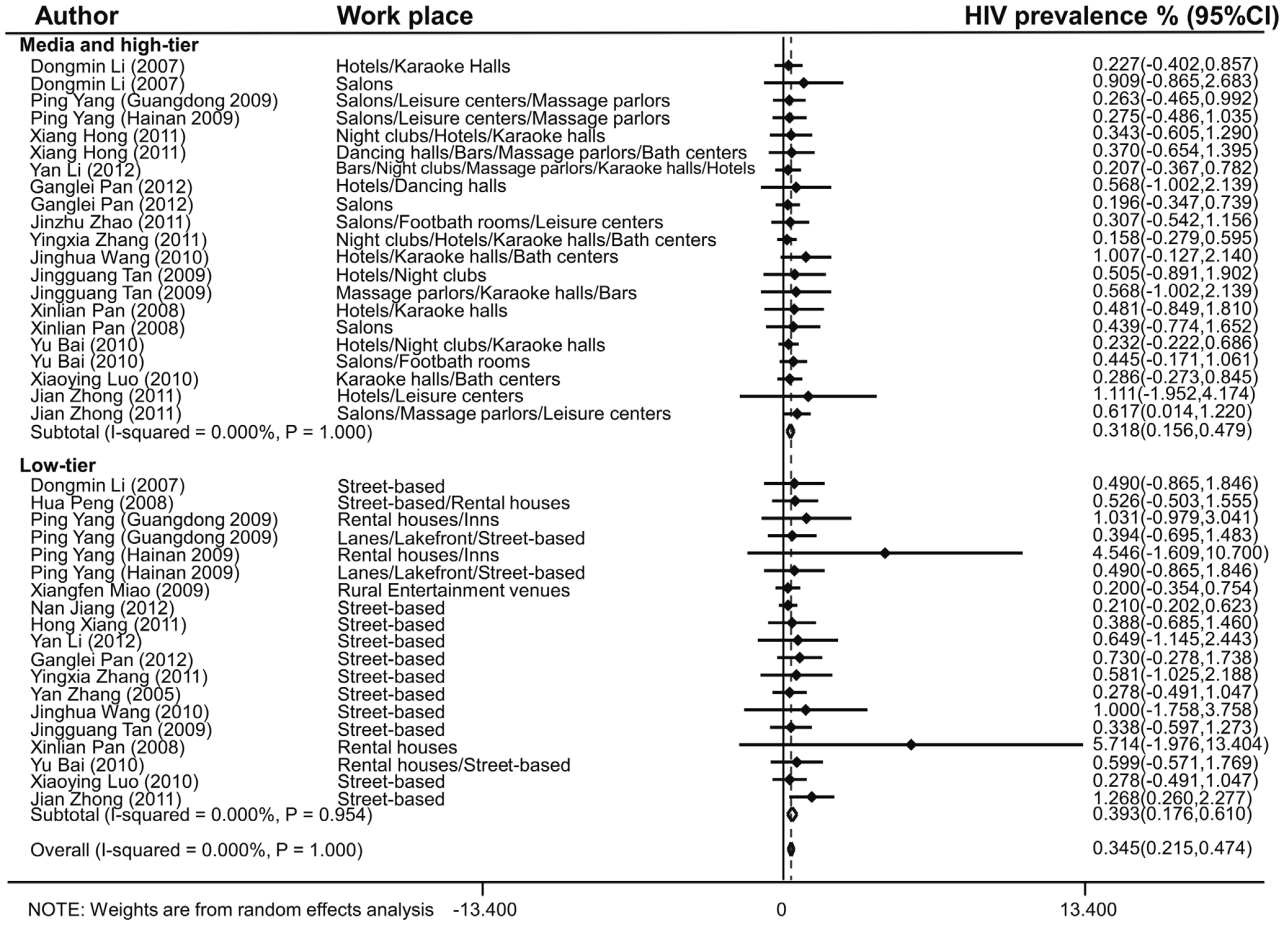
Forest plot showing estimated HIV prevalence in Chinese female sex workers in different workplaces. Workplaces were categorized into two grades: Medium and high-tier (Hotels, Karaoke halls, Salons, Leisure centers, Massage parlors, Night clubs, Dancing halls, Bars, Bath centers and Footbath rooms) and Low-tier (Street-based, Rental houses, Inns, Lanes, Lakefront and Rural entertainment venues).

**Figure 4:**
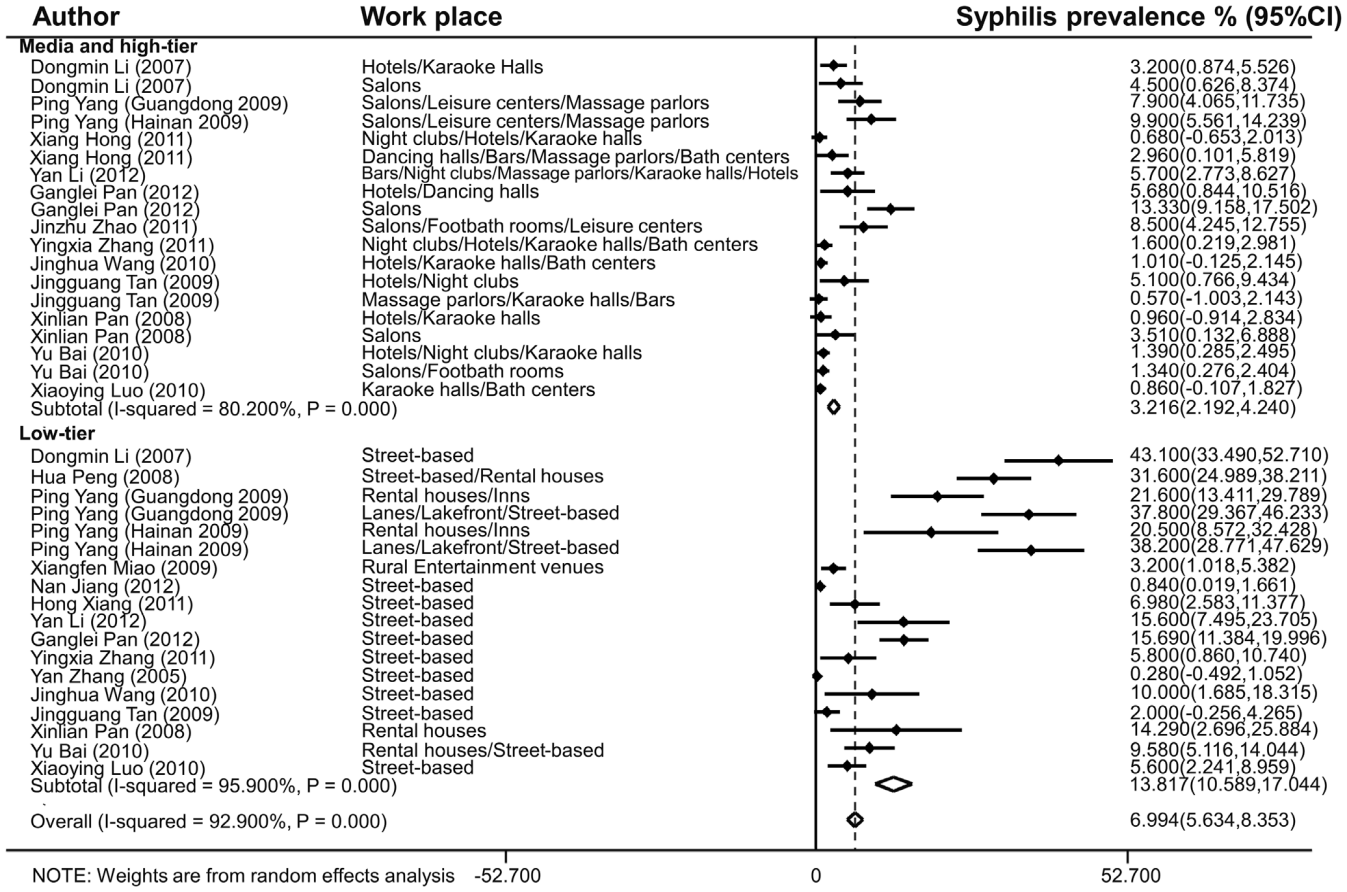
Forest plot showing estimated syphilis prevalence in Chinese female sex workers in different workplaces. Workplaces were categorized into two grades: Medium and high-tier (Hotels, Karaoke halls, Salons, Leisure centers, Massage parlors, Night clubs, Dancing halls, Bars, Bath centers and Footbath rooms) and Low-tier (Street-based, Rental houses, Inns, Lanes, Lakefront and Rural entertainment venues).

The estimated HIV prevalence in the medium and high-tier, and low-tier workplaces was 0.318% (95% CI: 0.156–0.479%) and 0.393% (95% CI: 0.176–0.610%), respectively (Figure 3). There was no heterogeneity for the studies reporting HIV prevalence (Q test *p* = 1.000, *I* ^2^ = 0.000%). The estimated syphilis prevalence was 3.216% (95% CI: 2.192–4.240%) in medium and high-tier workplaces, and 13.817% (95% CI: 10.589–17.044%) in low-tier workplaces (Figure 4). And heterogeneity (Q test *p* = 0.000, *I* ^2^ = 92.900%) were observed.

## Discussion

Data from our study suggested a decline in HIV and syphilis epidemics during the period from 2000 to 2011 in Chinese FSWs on a national level. Both HIV and syphilis prevalence showed regional discrepancies. FSWs in low-tier workplaces may be at higher risk of HIV and syphilis infection than those in high-tier workplaces. To the best of our knowledge, our study is the first to explore the chronological epidemic trends and regional and workplace discrepancies in HIV and syphilis infection among FSWs in mainland China by systematic review and meta-analysis.

China's epidemiological surveillance showed an evident upward trend in HIV epidemics in FSWs before 2001, but there were significant fluctuations between 2001 and 2009 [10]. One reason for the difficulty in ascertaining any trends in HIV epidemics in Chinese FSWs is that HIV prevalence in this population has been relatively low and stable in China [7]. By meta-analysis and further by correlation analysis, we were able to demonstrate a downward trend in HIV prevalence between 2000 and 2011 in FSWs on a national level. However, our most recent estimated HIV prevalence was 0.041% (2009–2011), lower than the estimated HIV prevalence of 3.000% (2007–2011) in another meta-analysis [8]. A possible explanation is that the meta-analysis conducted by Doctor Baral and colleagues only included articles from China published in English (not in Chinese); there were 12 studies included in their meta-analysis (Yunnan, 6, Guangxi, 1, Guangdong, 2, Zhejiang, 1, Hong Kong, 1, and Henan, 1), among which five studies were also included in our research (Guangxi, 1, Yunnan, 2, Zhejiang, 1, and Guangdong, 1); and the 12 studies in Doctor Baral and colleagues' meta-analysis were mainly from provinces (Yunnan, Guangxi, Guangdong and Zhejiang) which had a higher pooled HIV prevalence in our study. In our meta-analysis the pooled HIV prevalence of studies published in Chinese and English was 0.171% and 2.430%, respectively, revealing national and international variation as discussed by Baral et al.

In addition, surveillance data showed median syphilis prevalence in FSWs fluctuated between 0.5% and 1.8% from 2004 to 2008 within 15 cities in different provinces of China [11]. Much information can be lost by estimating prevalence through medians. The most recent estimated syphilis prevalence in our study was 3.169% (95% CI: 2.738–3.600%) in the period from 2009 to 2011. Longitudinal studies on syphilis infection among FSWs in China have seldom been undertaken. A few studies showed a decline in syphilis prevalence in FSWs in different Chinese regions, such as Guangzhou, between 2002 and 2005 [12]. Another systematic review conducted in 2005 suggested syphilis prevalence increased at a rate of 1.4% per year among incarcerated FSWs during the period from 2000 to 2005 [13]. Coincidently, our data suggested an upward trend in syphilis prevalence in FSWs in Reeducation Centers that were places where female sex workers were administratively detained for moral and political “re-education” and vocational training (as shown in Table S6, although Table 2 shows that *r_s_* was not statistically significant). However, downward trends were seen in the other two subgroups, Entertainment venues and Others (mainly from medical institutions and STD clinics), that were under the health surveillance system. In China, tests for STDs in incarcerated FSWs or those in reeducation centers are obligatory. These facts suggested that the health surveillance system might not cover the very high-risk FSWs, or that some FSWs with STDs might intentionally avoid health surveillance because of fear of humiliation or for other reasons.

Heterosexual transmission as the main driver of HIV infection rates in China seems contradictory to the decline in HIV prevalence in FSWs. Several possible reasons for this are evident from a previous report [14]. Firstly, among all individuals infected with HIV through heterosexual transmission, about one-third were infected by their spouses and two-thirds were not, and in the latter group some were infected through non-commercial sexual behaviors (sexual behaviors without the direct purpose of money, such as extramarital affairs, “one-night stands” and having many sexual partners before marriage). Non-commercial sexual behaviors may therefore be an important route for heterosexual HIV transmission in China. Secondly, due to various limitations such as simple sampling methods and the FSWs' avoidance from surveillance, the system of surveillance might not cover the very high-risk FSWs who transmitted HIV. Thirdly, the working time of Chinese FSWs in the sex industry was about 5–6 years, meaning that about one-fifth of all FSWs were replaced every year. This turnover diluted HIV prevalence in FSWs.

A specific epidemiological character of HIV epidemics is the presence of regional disparities based on the different routes of transmission in China. Border regions (Yunnan, Guangxi and Xinjiang) had a higher HIV incidence among IDUs, in coastal areas (Guangdong and Fujian) most HIV infections were sexually transmitted, and in central China (Henan, Anhui, Hubei and Shanxi) HIV-infected people were mainly FPDs [15]. Studies from Guangzhou and Yunnan showed that 45% to 100% of HIV-infected FSWs reported a history of intravenous drug use and 12% to 49% of FSWs who were IDUs were HIV positive [7]. Meanwhile, FSWs in these provinces might also have had more clients who were drug users. So the fact that some FSWs are also IDUs may have contributed to larger differences between HIV and syphilis prevalence in some provinces, such as Yunnan, than in others.

Data on FSWs in lower-tier work settings were limited because of the difficulties in approaching them. For this reason, and also the low HIV prevalence among Chinese FSWs, our data suggested minor differences in HIV prevalence in different workplaces, although they showed evident differences in syphilis prevalence. FSWs in lower-tier work settings may be older, more illiterate and less aware of HIV/STD, may have worked for much longer in the sex industry, and may have a much lower economic status, more high-risk sexual behaviors and more clients of low income compared to those in higher tier work settings [16], [17]. More efforts and studies are needed in this group to find more effective ways of investigation and intervention, to reveal the very HIV/STD epidemic condition, and to enhance their awareness of HIV/STD. And this group is also an important focus for the future, in order to ensure they are included in the overall HIV/STD prevention and control strategies in China.

Several limitations should be noted in our study. Studies using nontreponemal or treponemal tests to diagnose syphilis infection were all included in our meta-analysis, which as a result, might have overestimated the current prevalence of syphilis, but underestimated the extent of decline in syphilis prevalence over time. Given the downward trend in syphilis prevalence shown in our study and others [12], the actual extent to which syphilis is declining may be greater. In the subgroup analysis (Table 2 and Table S6) our data further suggested a downward trend in syphilis epidemics in all three subgroups (the Treponemal tests group, the Nontreponemal tests group, and the Unspecified group). Secondly, there was a possibility of some selection bias. To assess the relationship between HIV and syphilis prevalence, we only included studies testing HIV and syphilis in the same cohorts, so information in the excluded studies only targeting either HIV or syphilis was missing. We only included peer-reviewed journal articles and so some unpublished data or reports from non-governmental organizations were missing, which is also likely to have contributed to selection bias. Thirdly, an unbalanced distribution of datasets and sample sizes between different subgroups over the study period (2000–2011) might be an important confounder in the investigation of HIV/syphilis epidemic trends, since subgroup analysis suggested discrepancies between subgroups (Table S5). Because of the complexity within the included studies (too many stratified factors and relatively limited datasets) we did not use a completely stratified analysis. Instead we investigated the HIV/syphilis epidemic trends in each subgroup (Table 2 and Table S6) and the distribution of the datasets and sample size among subgroups and study time periods (Table S7). Table 2 shows that all the *r_s_* that were statistically significant suggested a downward trend in HIV/syphilis epidemics. Table S7 shows that the higher numbers of datasets and greater sample sizes in the subgroups with a higher pooled prevalence of HIV (such as Language – English, Sample size <200, Geographical location – Southwest, and Sampling methods – Convenience sampling) and syphilis (such as Language – English, Sample size <200, Geographical location – South China and East China, and Syphilis testing methods – Treponemal tests) occurred mainly in the later period (2006–2011). These supplementary data therefore also suggested a downward trend in HIV and syphilis epidemics over the study period. Fourthly, different testing methods have different sensitivity and specificity. As can be seen in Table S6, combined rate from different testing methods were variable, for both HIV and syphilis. So pooling rate from different testing methods might bring bias. A single protocol using testing methods with high sensitivity and specificity is suggested in the future studies and surveillance systems.

In China, HIV/STD is not only a public health but also a socio-economic and political issue. Guided by information from the national surveillance system, China's HIV/STD policies were developed, including “Four Frees and One Care” (free anti-retroviral drugs to AIDS patients who are rural residents or people without insurance in urban areas; free voluntary counselling and testing; free anti-retroviral drugs to HIV-infected pregnant women, and HIV testing of newborn babies; free schooling for AIDS orphans and children from HIV infected families; and care and economic assistance to the households of PLHIV), 5-year action plans, and HIV/AIDS regulation, which were the landmark national level policies that have had a major impact on China's HIV/AIDS response. These national policies contained comprehensive measures in response to HIV/STD epidemics, including prevention and education policies, trearment measures and policies, care policies, and so on. The decline in HIV and syphilis prevalence in Chinese FSWs between 2000 and 2011 suggested in our study indicates the achievements not only in the 2000s, but also in the 1990s, given that the effects of the plans might only be apparent after several years. However, it does not mean that HIV and syphilis epidemics are now under control in China and there remain some challenges such as discrimination hindering the effective prevention and control of HIV/STD, limitations in the healthcare system with insufficient coverage of testing, treatment and prevention services and limited financial support. Thus funding and attention is still needed in the future. And strong commitment and a problem solving-oriented approach will allow China to better address and cope with these challenges.

## Supporting Information

Table S1 **PRISMA 2009 checklist.**
(DOC)

Table S2 **Search strategy for PubMed search.**
(DOC)

Table S3 **Data extraction table of 190 included studies with 341 records.**
(DOC)

Table S4 **Data extraction table of Chinese female sex workers in different workplaces.**
(DOC)

Table S5 **Results of subgroup analysis and meta-regression.**
(DOC)

Table S6 **HIV and syphilis epidemic trends by subgroup in each three-year study period.**
(DOC)

Table S7 **Distribution of HIV/syphilis prevalence datasets and sample sizes between subgroups and the four three-year study periods.**
(DOC)
