# Supplementary material for: A Decline in HIV and Syphilis Epidemics in Chinese Female Sex Workers (2000–2011): A Systematic Review and Meta-Analysis
Source: PLoS One. 2013 Dec 13;8(12):e82451. doi: 10.1371/journal.pone.0082451 (PMC3862622; doi:10.1371/journal.pone.0082451)
Supplement: Table S2 — Search strategy for PubMed search. (DOC) [file pone.0082451.s002.doc]

**Table S2**. Search strategy for PubMed search.

| **Order** | **Search strategy**^*^ |
| --- | --- |
| #1) | “HIV”[Mesh] |
| #2) | HIV |
| #3) | “Acquired Immunodeficiency Syndrome”[Mesh] |
| #4) | AIDS |
| #5) | #1) OR #2) OR #3) OR #4) |
| #6) | “Sexually Transmitted Diseases”[Mesh] |
| #7) | sexually transmitted diseases |
| #8) | sexually transmitted infection |
| #9) | STI |
| #10) | “Syphilis”[Mesh] |
| #11) | syphilis |
| #12) | #6) OR #7) OR #8) OR #9) OR #10) OR #11) |
| #13) | “Prostitution”[Mesh] |
| #14) | prostitution |
| #15) | “Sex Workers”[Mesh] |
| #16) | sex worker |
| #17) | sex work |
| #18) | sex work* |
| #19) | female sex worker |
| #20) | commercial sex worker |
| #21) | #13) OR #14) OR #15) OR #16) OR #17) OR #18) OR #19) OR #20) |
| #22) | “China”[Mesh] |
| #23) | china |
| #24) | #22) OR #23) |
| #25) | #5) AND #12) AND #21) AND #24) |
| #26) | #5) AND #12) AND #21) AND #24) Filters: English; Chinese |

^*^ “”, exact search; [Mesh], Medical Subject Headings; OR/AND, Boolean logistic operator; *, truncation search.
