# Supplementary material for: A Decline in HIV and Syphilis Epidemics in Chinese Female Sex Workers (2000–2011): A Systematic Review and Meta-Analysis
Source: PLoS One. 2013 Dec 13;8(12):e82451. doi: 10.1371/journal.pone.0082451 (PMC3862622; doi:10.1371/journal.pone.0082451)
Supplement: Table S3 — Data extraction table of 190 included studies with 341 records. (DOC) [file pone.0082451.s003.doc]

**Table S3.** Data extraction table of 190 included studies with 341 records.

| **First author**^‡‡^ | **Year of publication** | **Language** | **Study period**^*^ | **Province**^†^ | **Study design** | **Study location**^‡^ | **Sampling methods** | **HIV testing**  **methods**^**^ | **Syphilis**  **testing methods**^††^ | **Sample size**^§^ | **HIV**  **testing cases** | **Prevalence of HIV %** | **Syphilis testing cases** | **Prevalence of syphilis %** |
| --- | --- | --- | --- | --- | --- | --- | --- | --- | --- | --- | --- | --- | --- | --- |
| Wen Xiaoqing^1^ | 2009 | Chinese | 2007 | Guangxi | Cross-sectional Study | Entertainment Venues | Stratified Random Sampling | ELISA | RPR | 360 | 360 | 0.00 | 360 | 1.39 |
| Li Dongmin^2^ | 2007 | Chinese | 2006 | Guizhou | Cross-sectional Study | Entertainment Venues | Stratified Random Sampling | ELISA-1/ELISA-2 | RPR/TPPA | 432 | 432 | 0.20 | 432 | 13.00 |
| Hang Hong^3^ | 2010 | English | 2006 | Zhejiang | Intervention Study | Entertainment Venues | Convenience Sampling | ELISA/WB | RPR/TPPA | 403 | 403 | 0.40 | 403 | 13.50 |
| Xu Yajuan^4^ | 2011 | Chinese | 2009 | Jiangxi | Cross-sectional Study | Entertainment Venues | Convenience Sampling | ELISA-1/ELISA-2/WB | RPR/ELISA | 5514 | 5513 | 0.05 | 5513 | 0.73 |
| Nie Zhiqiang^5^ | 2011 | Chinese | 2009 | Guangdong | Cross-sectional Study | Entertainment Venues | Stratified Random Sampling | ELISA | RPR | 5309 | 5308 | 0.20 | 5285 | 3.10 |
| Zhong Jian^6^ | 2011 | Chinese | 2009 | Guangxi | Cross-sectional Study | Entertainment Venues | Stratified Random Sampling | ELISA | TRUST | None^¶^ | 1166 | 0.86 | 1007 | 0.99 |
| Guo Yuanyu^7^ | 2010 | Chinese | 2001 | Zhejiang | Cross-sectional Study | Reeducation Center | Convenience Sampling | ELISA/WB | RPR/TPHA | None^¶^ | 331 | 0.00 | 331 | 12.70 |
|  |  |  | 2002 | Zhejiang | Cross-sectional Study | Reeducation Center | Convenience Sampling |  |  |  | 189 | 0.00 | 189 | 5.80 |
|  |  |  | 2003 | Zhejiang | Cross-sectional Study | Reeducation Center | Convenience Sampling |  |  |  | 183 | 0.00 | 183 | 7.70 |
|  |  |  | 2004 | Zhejiang | Cross-sectional Study | Reeducation Center | Convenience Sampling |  |  |  | 319 | 0.00 | 319 | 7.20 |
|  |  |  | 2005 | Zhejiang | Cross-sectional Study | Reeducation Center | Convenience Sampling |  |  |  | 279 | 0.00 | 279 | 5.70 |
|  |  |  | 2006 | Zhejiang | Cross-sectional Study | Reeducation Center | Convenience Sampling |  |  |  | 212 | 0.00 | 212 | 8.00 |
|  |  |  | 2007 | Zhejiang | Cross-sectional Study | Reeducation Center | Convenience Sampling |  |  |  | 160 | 0.00 | 160 | 7.50 |
| Han Wenxiang^8^ | 2012 | Chinese | 2007 | Yunnan | Cross-sectional Study | Entertainment Venues | Convenience Sampling | Unspecified | Unspecified |  | 137 | 6.57 | 137 | 3.65 |
|  |  |  | 2008 | Yunnan | Cross-sectional Study | Entertainment Venues | Convenience Sampling |  |  |  | 121 | 4.13 | 121 | 1.65 |
|  |  |  | 2009 | Yunnan | Cross-sectional Study | Entertainment Venues | Convenience Sampling |  |  |  | 128 | 1.56 | 128 | 0.00 |
| Yang Meixia^9^ | 2009 | Chinese | 2005 | Shanghai | Cross-sectional Study | Entertainment Venues | Convenience Sampling | Unspecified | RPR/TPPA |  | 79 | 0.00 | 79 | 6.30 |
| Wang Yingxin^10^ | 2011 | Chinese | 2009 | Shandong | Cross-sectional Study | Entertainment Venues | Stratified Random Sampling | ELISA | ELISA/TPPA | 4641 | 4641 | 0.00 | 4641 | 2.20 |
| Wang Lan^11^ | 2009 | Chinese | 2004 | Anhui | Cross-sectional Study | Entertainment Venues | Convenience Sampling | ELISA-1/ELISA-2 | RPR | 373 | 373 | 0.00 | 373 | 0.50 |
|  |  |  | 2004 | Shenzhen,  Guangdong | Cross-sectional Study | Entertainment Venues | Convenience Sampling |  |  | 371 | 371 | 0.00 | 371 | 6.50 |
|  |  |  | 2004 | Zhuhai,  Guangdong | Cross-sectional Study | Entertainment Venues | Convenience Sampling |  |  | 325 | 325 | 0.00 | 325 | 2.50 |
|  |  |  | 2004 | Guangxi | Cross-sectional Study | Entertainment Venues | Convenience Sampling |  |  | 360 | 360 | 0.00 | 360 | 10.60 |
|  |  |  | 2004 | Hubei | Cross-sectional Study | Entertainment Venues | Convenience Sampling |  |  | 369 | 369 | 0.00 | 369 | 3.00 |
|  |  |  | 2004 | Hunan | Cross-sectional Study | Entertainment Venues | Convenience Sampling |  |  | 124 | 124 | 0.00 | 124 | 0.00 |
|  |  |  | 2004 | Jilin | Cross-sectional Study | Entertainment Venues | Convenience Sampling |  |  | 314 | 314 | 0.00 | 314 | 0.30 |
|  |  |  | 2004 | Jiangsu | Cross-sectional Study | Entertainment Venues | Convenience Sampling |  |  | 366 | 366 | 0.00 | 366 | 3.00 |
|  |  |  | 2004 | Liaoning | Cross-sectional Study | Entertainment Venues | Convenience Sampling |  |  | 379 | 379 | 0.00 | 379 | 0.50 |
|  |  |  | 2004 | Ningxia | Cross-sectional Study | Entertainment Venues | Convenience Sampling |  |  | 326 | 326 | 0.30 | 326 | 0.00 |
|  |  |  | 2004 | Shanxi | Cross-sectional Study | Entertainment Venues | Convenience Sampling |  |  | 360 | 360 | 0.30 | 360 | 2.80 |
|  |  |  | 2004 | Shanghai | Cross-sectional Study | Entertainment Venues | Convenience Sampling |  |  | 314 | 314 | 0.00 | 314 | 0.30 |
|  |  |  | 2004 | Xinjiang | Cross-sectional Study | Entertainment Venues | Convenience Sampling |  |  | 254 | 254 | 0.40 | 254 | 0.40 |
|  |  |  | 2005 | Anhui | Cross-sectional Study | Entertainment Venues | Convenience Sampling |  |  | 324 | 324 | 0.30 | 324 | 2.80 |
|  |  |  | 2005 | Shenzhen,  Guangdong | Cross-sectional Study | Entertainment Venues | Convenience Sampling |  |  | 354 | 354 | 0.00 | 354 | 2.80 |
|  |  |  | 2005 | Zhuhai,  Guangdong | Cross-sectional Study | Entertainment Venues | Convenience Sampling |  |  | 204 | 204 | 0.00 | 204 | 0.00 |
|  |  |  | 2005 | Guangxi | Cross-sectional Study | Entertainment Venues | Convenience Sampling |  |  | 385 | 385 | 0.00 | 385 | 15.30 |
|  |  |  | 2005 | Hainan | Cross-sectional Study | Entertainment Venues | Convenience Sampling |  |  | 148 | 148 | 0.00 | 148 | 4.70 |
|  |  |  | 2005 | Hubei | Cross-sectional Study | Entertainment Venues | Convenience Sampling |  |  | 351 | 351 | 0.00 | 351 | 0.90 |
|  |  |  | 2005 | Hunan | Cross-sectional Study | Entertainment Venues | Convenience Sampling |  |  | 399 | 399 | 0.00 | 399 | 3.50 |
|  |  |  | 2005 | Jilin | Cross-sectional Study | Entertainment Venues | Convenience Sampling |  |  | 361 | 361 | 0.00 | 361 | 0.60 |
|  |  |  | 2005 | Jiangsu | Cross-sectional Study | Entertainment Venues | Convenience Sampling |  |  | 396 | 396 | 0.50 | 396 | 0.50 |
|  |  |  | 2005 | Liaoning | Cross-sectional Study | Entertainment Venues | Convenience Sampling |  |  | 420 | 420 | 0.00 | 420 | 0.00 |
|  |  |  | 2005 | Ningxia | Cross-sectional Study | Entertainment Venues | Convenience Sampling |  |  | 360 | 360 | 0.00 | 360 | 1.10 |
|  |  |  | 2005 | Shandong | Cross-sectional Study | Entertainment Venues | Convenience Sampling |  |  | 251 | 251 | 0.00 | 251 | 2.40 |
|  |  |  | 2005 | Shanxi | Cross-sectional Study | Entertainment Venues | Convenience Sampling |  |  | 363 | 363 | 0.00 | 363 | 0.60 |
|  |  |  | 2005 | Shanghai | Cross-sectional Study | Entertainment Venues | Convenience Sampling |  |  | 278 | 278 | 0.40 | 278 | 10.40 |
|  |  |  | 2005 | Xinjiang | Cross-sectional Study | Entertainment Venues | Convenience Sampling |  |  | 331 | 331 | 1.20 | 331 | 1.80 |
|  |  |  | 2006 | Anhui | Cross-sectional Study | Entertainment Venues | Convenience Sampling |  |  | 132 | 132 | 0.00 | 132 | 4.50 |
|  |  |  | 2006 | Shenzhen,  Guangdong | Cross-sectional Study | Entertainment Venues | Convenience Sampling |  |  | 76 | 76 | 0.00 | 76 | 5.30 |
|  |  |  | 2006 | Zhuhai,  Guangdong | Cross-sectional Study | Entertainment Venues | Convenience Sampling |  |  | 80 | 80 | 1.30 | 80 | 1.30 |
|  |  |  | 2006 | Guangxi | Cross-sectional Study | Entertainment Venues | Convenience Sampling |  |  | 106 | 106 | 0.00 | 106 | 4.70 |
|  |  |  | 2006 | Hubei | Cross-sectional Study | Entertainment Venues | Convenience Sampling |  |  | 215 | 215 | 0.50 | 215 | 2.30 |
|  |  |  | 2006 | Hunan | Cross-sectional Study | Entertainment Venues | Convenience Sampling |  |  | 75 | 75 | 0.00 | 75 | 0.00 |
|  |  |  | 2006 | Jilin | Cross-sectional Study | Entertainment Venues | Convenience Sampling |  |  | 217 | 217 | 0.00 | 217 | 1.80 |
|  |  |  | 2006 | Jiangsu | Cross-sectional Study | Entertainment Venues | Convenience Sampling |  |  | 49 | 49 | 0.00 | 49 | 6.10 |
|  |  |  | 2006 | Liaoning | Cross-sectional Study | Entertainment Venues | Convenience Sampling |  |  | 242 | 242 | 0.00 | 242 | 0.00 |
|  |  |  | 2006 | Ningxia | Cross-sectional Study | Entertainment Venues | Convenience Sampling |  |  | 167 | 167 | 0.00 | 167 | 1.20 |
|  |  |  | 2006 | Shandong | Cross-sectional Study | Entertainment Venues | Convenience Sampling |  |  | 67 | 67 | 0.00 | 67 | 0.00 |
|  |  |  | 2006 | Shanxi | Cross-sectional Study | Entertainment Venues | Convenience Sampling |  |  | 199 | 199 | 0.00 | 199 | 0.50 |
|  |  |  | 2006 | Shanghai | Cross-sectional Study | Entertainment Venues | Convenience Sampling |  |  | 221 | 221 | 0.00 | 221 | 39.80 |
|  |  |  | 2006 | Xinjiang | Cross-sectional Study | Entertainment Venues | Convenience Sampling |  |  | 129 | 129 | 0.00 | 129 | 0.00 |
|  |  |  | 2007 | Anhui | Cross-sectional Study | Entertainment Venues | Convenience Sampling |  |  | 381 | 381 | 0.00 | 381 | 13.40 |
|  |  |  | 2007 | Shenzhen,  Guangdong | Cross-sectional Study | Entertainment Venues | Convenience Sampling |  |  | 343 | 343 | 0.00 | 343 | 3.80 |
|  |  |  | 2007 | Zhuhai,  Guangdong | Cross-sectional Study | Entertainment Venues | Convenience Sampling |  |  | 322 | 322 | 0.00 | 322 | 1.60 |
|  |  |  | 2007 | Guangxi | Cross-sectional Study | Entertainment Venues | Convenience Sampling |  |  | 454 | 454 | 0.00 | 454 | 3.30 |
|  |  |  | 2007 | Hubei | Cross-sectional Study | Entertainment Venues | Convenience Sampling |  |  | 410 | 410 | 0.50 | 410 | 5.40 |
|  |  |  | 2007 | Hunan | Cross-sectional Study | Entertainment Venues | Convenience Sampling |  |  | 321 | 321 | 0.00 | 321 | 3.70 |
|  |  |  | 2007 | Jilin | Cross-sectional Study | Entertainment Venues | Convenience Sampling |  |  | 401 | 401 | 0.00 | 401 | 0.70 |
|  |  |  | 2007 | Jiangsu | Cross-sectional Study | Entertainment Venues | Convenience Sampling |  |  | 394 | 394 | 0.00 | 394 | 1.30 |
|  |  |  | 2007 | Liaoning | Cross-sectional Study | Entertainment Venues | Convenience Sampling |  |  | 396 | 396 | 0.00 | 396 | 0.50 |
|  |  |  | 2007 | Ningxia | Cross-sectional Study | Entertainment Venues | Convenience Sampling |  |  | 424 | 424 | 0.00 | 424 | 0.70 |
|  |  |  | 2007 | Shandong | Cross-sectional Study | Entertainment Venues | Convenience Sampling |  |  | 399 | 399 | 0.00 | 399 | 1.30 |
|  |  |  | 2007 | Shanxi | Cross-sectional Study | Entertainment Venues | Convenience Sampling |  |  | 496 | 496 | 0.00 | 496 | 0.20 |
|  |  |  | 2007 | Shanghai | Cross-sectional Study | Entertainment Venues | Convenience Sampling |  |  | 253 | 253 | 0.00 | 253 | 28.50 |
|  |  |  | 2007 | Xinjiang | Cross-sectional Study | Entertainment Venues | Convenience Sampling |  |  | 400 | 400 | 0.80 | 400 | 2.00 |
|  |  |  | 2008 | Anhui | Cross-sectional Study | Entertainment Venues | Convenience Sampling |  |  | 402 | 402 | 0.00 | 402 | 2.70 |
|  |  |  | 2008 | Shenzhen,  Guangdong | Cross-sectional Study | Entertainment Venues | Convenience Sampling |  |  | 332 | 332 | 0.00 | 332 | 2.40 |
|  |  |  | 2008 | Zhuhai,  Guangdong | Cross-sectional Study | Entertainment Venues | Convenience Sampling |  |  | 493 | 493 | 0.00 | 493 | 0.40 |
|  |  |  | 2008 | Guangxi | Cross-sectional Study | Entertainment Venues | Convenience Sampling |  |  | 447 | 447 | 0.40 | 447 | 1.30 |
|  |  |  | 2008 | Hainan | Cross-sectional Study | Entertainment Venues | Convenience Sampling |  |  | 292 | 292 | 0.30 | 292 | 1.00 |
|  |  |  | 2008 | Hubei | Cross-sectional Study | Entertainment Venues | Convenience Sampling |  |  | 399 | 399 | 0.00 | 399 | 4.30 |
|  |  |  | 2008 | Hunan | Cross-sectional Study | Entertainment Venues | Convenience Sampling |  |  | 250 | 250 | 0.00 | 250 | 1.20 |
|  |  |  | 2008 | Jilin | Cross-sectional Study | Entertainment Venues | Convenience Sampling |  |  | 400 | 400 | 0.00 | 400 | 1.00 |
|  |  |  | 2008 | Jiangsu | Cross-sectional Study | Entertainment Venues | Convenience Sampling |  |  | 405 | 405 | 0.00 | 405 | 0.00 |
|  |  |  | 2008 | Liaoning | Cross-sectional Study | Entertainment Venues | Convenience Sampling |  |  | 400 | 400 | 0.00 | 400 | 0.50 |
|  |  |  | 2008 | Ningxia | Cross-sectional Study | Entertainment Venues | Convenience Sampling |  |  | 398 | 398 | 0.00 | 398 | 0.30 |
|  |  |  | 2008 | Shandong | Cross-sectional Study | Entertainment Venues | Convenience Sampling |  |  | 396 | 396 | 0.00 | 396 | 7.30 |
|  |  |  | 2008 | Shanxi | Cross-sectional Study | Entertainment Venues | Convenience Sampling |  |  | 400 | 400 | 0.00 | 400 | 0.50 |
|  |  |  | 2008 | Shanghai | Cross-sectional Study | Entertainment Venues | Convenience Sampling |  |  | 250 | 250 | 0.40 | 250 | 32.40 |
|  |  |  | 2008 | Xinjiang | Cross-sectional Study | Entertainment Venues | Convenience Sampling |  |  | 391 | 391 | 0.30 | 391 | 0.50 |
| Tang Zhanli^12^ | 2011 | Chinese | 2010 | Shandong | Cross-sectional Study | Entertainment Venues | Convenience Sampling | ELISA | RPR/TPPA | 322 | 322 | 0.00 | 322 | 6.20 |
| Li Mingqiang^13^ | 2010 | Chinese | 2008 | Guangxi | Cross-sectional Study | Unspecified | Convenience Sampling | ELISA+WB | RPR | 448 | 448 | 0.40 | 448 | 1.30 |
| Peng Hua^14^ | 2008 | Chinese | 2007 | Guangdong | Cross-sectional Study | Entertainment Venues | Convenience Sampling | Immunocolloidal gold/WB | TRUST/TPPA | 190 | 190 | 0.50 | 190 | 31.60 |
| Yang Ping^15^ | 2009 | Chinese | 2007 | Guangdong | Cross-sectional Study | Entertainment Venues | Convenience Sampling | Immunocolloidal gold/WB | TRUST/TPPA | 414 | 414 | 0.25 | 414 | 20.29 |
|  |  |  | 2007 | Hainan | Cross-sectional Study | Entertainment Venues | Convenience Sampling | Unspecified |  | 328 | 328 | 0.61 | 328 | 20.12 |
| Ouyang Hong^16^ | 2012 | Chinese | 2011 | Sichuan | Cross-sectional Study | Entertainment Venues | Convenience Sampling | ELISA-1/ELISA-2 | RPR | 400 | 400 | 0.25 | 400 | 2.75 |
| Pei Dongnu^17^ | 2002 | Chinese | 2001 | Hainan | Cross-sectional Study | Reeducation Center | Convenience Sampling | ELISA | RPR/TPPA | 317 | 317 | 0.00 | 317 | 17.67 |
| Wang Haibo^18^ | 2011 | English | 2007 | Yunnan | Cross-sectional Study | Entertainment Venues | Convenience Sampling | ELISA/WB | RPR/TPPA | 737 | 737 | 10.30 | 737 | 7.50 |
| Xu Junjie^19^ | 2011 | English | 2006 | Yunnan | Cohort Study | Entertainment Venues | Convenience Sampling | ELISA/WB | RPR/TPPA | 1642 | 1642 | 10.20 | 1642 | 8.30 |
| Luo Yan^20^ | 2008 | Chinese | 2007 | Zhejiang | Cross-sectional Study | Entertainment Venues | Convenience Sampling | ELISA/WB | RPR/TPPA | 267 | 267 | 0.00 | 267 | 2.60 |
| Jiang Yongjun^21^ | 2002 | Chinese | 2000 | Liaoning | Cross-sectional Study | Reeducation Center | Convenience Sampling | ELISA/WB | TPHA | 162 | 162 | 0.00 | 162 | 9.30 |
| Miao Xiangfen^22^ | 2009 | Chinese | 2008 | Hebei | Cross-sectional Study | Entertainment Venues | Convenience Sampling | ELISA | RPR/TPPA | 250 | 250 | 0.00 | 250 | 3.20 |
| Jiang Nan^23^ | 2012 | Chinese | 2010 | Henan | Cross-sectional Study | Entertainment Venues and Reeducation Center | Convenience Sampling | ELISA/WB | RPR/TPPA | 475 | 475 | 0.21 | 475 | 0.84 |
| Chen Guosheng^24^ | 2010 | Chinese | 2009 | Jiangxi | Cross-sectional Study | Entertainment Venues | Convenience Sampling | ELISA/WB | RPR/TPPA | 401 | 401 | 0.00 | 401 | 2.00 |
| Zhao Gaoding^25^ | 2011 | Chinese | 2010 | Shaanxi | Cross-sectional Study | Entertainment Venues | Stratified Random Sampling | ELISA | ELISA | 412 | 412 | 0.00 | 412 | 1.94 |
| Zhang Qian^26^ | 2008 | Chinese | 2007 | Anhui | Cross-sectional Study | Entertainment Venues | Convenience Sampling | ELISA/WB | ELISA | 400 | 394 | 0.00 | 394 | 13.10 |
| Zhang Mingning^27^ | 2011 | Chinese | 2010 | Shanxi | Cross-sectional Study | Entertainment Venues | Convenience Sampling | Unspecified | Unspecified | 400 | 400 | 0.00 | 400 | 4.80 |
| Zhang Li^28^ | 2011 | Chinese | 2009 | Henan | Cross-sectional Study | Entertainment Venues | Convenience Sampling | Unspecified | Unspecified | 410 | 395 | 0.00 | 395 | 2.00 |
| Zhang Hong^29^ | 2011 | Chinese | 2010 | Fujian | Cross-sectional Study | Unspecified | Convenience Sampling | ELISA-1/ELISA-2 | RPR/ELISA | 479 | 479 | 0.00 | 479 | 0.21 |
| Yi Jianfei^30^ | 2010 | Chinese | 2009 | Jiangxi | Cross-sectional Study | Entertainment Venues | Convenience Sampling | ELISA/WB | RPR | 3868 | 3868 | 0.03 | 3868 | 1.81 |
| Yan Wenzuo^31^ | 2010 | Chinese | 2009 | Yunnan | Cross-sectional Study | Entertainment Venues | Convenience Sampling | ELISA/WB | ELISA | 188 | 188 | 1.10 | 188 | 6.40 |
| Xu Xiangyun^32^ | 2011 | Chinese | 2009 | Inner Mongolia | Cross-sectional Study | Entertainment Venues | Convenience Sampling | ELISA/WB | Unspecified | 400 | 400 | 0.25 | 400 | 5.50 |
| Xiang Hong^33^ | 2011 | Chinese | 2009 | Hubei | Cross-sectional Study | Entertainment Venues | Stratified Random Sampling | ELISA | RPR | 400 | 400 | 0.00 | 400 | 3.50 |
| Wu Jun^34^ | 2012 | Chinese | 2011 | Anhui | Cross-sectional Study | Entertainment Venues | Convenience Sampling | Unspecified | TRUST/ELISA | 405 | 405 | 0.00 | 405 | 9.63 |
| Wei Wei^35^ | 2011 | Chinese | 2010 | Henan | Cross-sectional Study | Entertainment Venues | Convenience Sampling | ELISA-1/ELISA-2 | RPR/ELISA | 405 | 405 | 0.25 | 405 | 1.23 |
| Wang Fang^36^ | 2010 | Chinese | 2009 | Anhui | Cross-sectional Study | Entertainment Venues | Convenience Sampling | Unspecified | Unspecified | 75 | 75 | 0.00 | 75 | 0.00 |
| Wang Dongli^37^ | 2012 | Chinese | 2011 | Jiangsu | Cross-sectional Study | Entertainment Venues | Convenience Sampling | ELISA-1/ELISA-2 | RPR | 400 | 400 | 0.25 | 400 | 2.00 |
| Tang Mengjin^38^ | 2012 | Chinese | 2011 | Guangxi | Cross-sectional Study | Entertainment Venues | Convenience Sampling | ELISA | RPR/TPPA | 1213 | 1213 | 0.80 | 1213 | 7.60 |
| Sun Xiaoqiang^39^ | 2011 | Chinese | 2010 | Anhui | Cross-sectional Study | Entertainment Venues | Convenience Sampling | ELISA | RPR | 354 | 354 | 0.00 | 354 | 1.10 |
| Shao Mengchi^40^ | 2010 | Chinese | 2009 | Jiangsu | Cross-sectional Study | Entertainment Venues | Convenience Sampling | ELISA/WB | RPR/TPPA | 402 | 402 | 0.00 | 402 | 3.70 |
| Qiu Zhihong^41^ | 2012 | Chinese | 2011 | Zhejiang | Cross-sectional Study | Entertainment Venues | Convenience Sampling | ELISA | RPR/TRUST/ELISA | 400 | 400 | 0.30 | 400 | 7.80 |
| Miao Xiaolan^42^ | 2011 | Chinese | 2010 | Jiangsu | Cross-sectional Study | Entertainment Venues and Reeducation Center | Convenience Sampling | ELISA-1/ELISA-2 | TRUST/ELISA | 800 | 800 | 0.00 | 800 | 4.60 |
| Luo Menghua^43^ | 2011 | Chinese | 2009 | Hunan | Cross-sectional Study | Entertainment Venues | Stratified Random Sampling | ELISA/WB | ELISA | 800 | 800 | 0.00 | 800 | 2.38 |
| Liao Meizhen^44^ | 2010 | Chinese | 2009 | Shandong | Cross-sectional Study | Entertainment Venues | Convenience Sampling | ELISA-1/ELISA-2 | RPR | 4732 | 4640 | 0.00 | 4637 | 2.18 |
| Li Yan^45^ | 2012 | Chinese | 2011 | Hubei | Cross-sectional Study | Entertainment Venues | Convenience Sampling | ELISA/WB | TRUST/ELISA | 400 | 400 | 0.25 | 400 | 3.00 |
| Jing Lihong^46^ | 2010 | Chinese | 2009 | Shanxi | Cross-sectional Study | Entertainment Venues | Convenience Sampling | ELISA-1/ELISA-2 | RPR/TPPA | 401 | 401 | 0.00 | 401 | 1.50 |
| Jiang Dingkang^47^ | 2010 | Chinese | 2009 | Shaanxi | Cross-sectional Study | Entertainment Venues and Reeducation Center | Convenience Sampling | ELISA | RPR/ELISA | 380 | 380 | 0.00 | 380 | 0.26 |
| Guo Changlu^48^ | 2010 | Chinese | 2009 | Jiangxi | Cross-sectional Study | Entertainment Venues | Convenience Sampling | ELISA/WB | RPR | 260 | 260 | 0.00 | 260 | 0.38 |
| Wen Xiaoqing^49^ | 2010 | Chinese | 2009 | Guangxi | Cross-sectional Study | Entertainment Venues | Convenience Sampling | ELISA | RPR | 400 | 400 | 0.25 | 400 | 2.00 |
| Nong Liping^50^ | 2011 | Chinese | 2010 | Guangxi | Cross-sectional Study | Entertainment Venues | Convenience Sampling | ELISA | TRUST | 177 | 177 | 0.58 | 177 | 2.30 |
| Li Fan^51^ | 2006 | Chinese | 2004 | Xinjiang | Cross-sectional Study | Entertainment Venues | Stratified Random Sampling | ELISA | RPR | 611 | 611 | 0.49 | 611 | 2.78 |
| Ying Yang^52^ | 2011 | English | 2008 | Shanghai | Cross-sectional Study | Entertainment Venues | Stratified Random Sampling | ELISA-1/ELISA-2/WB | TPHA | 411 | 411 | 0.00 | 411 | 7.00 |
|  |  |  | 2009 | Shanghai | Cross-sectional Study | Entertainment Venues | Stratified Random Sampling |  |  | 411 | 411 | 0.00 | 411 | 2.40 |
| Shi Junxia^53^ | 2012 | Chinese | 2009 | Guangdong | Intervention Study | Entertainment Venues | Convenience Sampling | ELISA/WB | RPR/ELISA/TPPA | 240 | 240 | 0.80 | 240 | 13.80 |
| Hu Bo^54^ | 2004 | Chinese | 2002 | Hainan | Intervention Study | Entertainment Venues | Convenience Sampling | Unspecified | Unspecified | 422 | 422 | 0.00 | 422 | 7.58 |
| He Jingchun^55^ | 2010 | Chinese | 2007 | Chongqin | Intervention Study | Entertainment Venues | Convenience Sampling | Unspecified | Unspecified | 417 | 417 | 0.00 | 417 | 1.20 |
| He Cai^56^ | 2009 | Chinese | 2008 | Guangdong | Cross-sectional Study | Entertainment Venues | Convenience Sampling | Unspecified | Unspecified | 335 | 335 | 0.00 | 335 | 2.40 |
| Xia Jianhui^57^ | 2010 | Chinese | 2000 | Tianjin | Cross-sectional Study | Reeducation Center | Convenience Sampling | ELISA/WB | RPR/TPPA |  | 620 | 0.16 | 620 | 7.10 |
|  |  |  | 2001 | Tianjin | Cross-sectional Study | Reeducation Center | Convenience Sampling |  |  |  | 648 | 0.00 | 648 | 6.94 |
|  |  |  | 2002 | Tianjin | Cross-sectional Study | Reeducation Center | Convenience Sampling |  |  |  | 678 | 0.00 | 678 | 7.08 |
|  |  |  | 2003 | Tianjin | Cross-sectional Study | Reeducation Center | Convenience Sampling |  |  |  | 407 | 0.00 | 407 | 7.13 |
|  |  |  | 2004 | Tianjin | Cross-sectional Study | Reeducation Center | Convenience Sampling |  |  |  | 607 | 0.16 | 607 | 4.45 |
|  |  |  | 2005 | Tianjin | Cross-sectional Study | Reeducation Center | Convenience Sampling |  |  |  | 315 | 0.00 | 315 | 4.13 |
|  |  |  | 2006 | Tianjin | Cross-sectional Study | Reeducation Center | Convenience Sampling |  |  |  | 449 | 0.45 | 449 | 5.57 |
|  |  |  | 2007 | Tianjin | Cross-sectional Study | Reeducation Center | Convenience Sampling |  |  |  | 178 | 0.00 | 178 | 11.24 |
|  |  |  | 2008 | Tianjin | Cross-sectional Study | Reeducation Center | Convenience Sampling |  |  |  | 148 | 0.68 | 148 | 6.76 |
| Yan Li^58^ | 2012 | English | 2007 | Guangdong | Cross-sectional Study | Medical Institutions | RDS||/snowball sampling | ELISA-1/ELISA-2/WB | RPR/TPHA | 320 | 318 | 0.00 | 318 | 8.18 |
| Luo Jie^59^ | 2005 | Chinese | 2003 | Guangxi | Cross-sectional Study | Entertainment Venues | Convenience Sampling | Unspecified | Unspecified | 312 | 312 | 1.08 | 312 | 4.67 |
| He Jiangang^60^ | 2005 | Chinese | 2004 | Anhui | Cross-sectional Study | Entertainment Venues | Stratified Random Sampling | ELISA | RPR | 400 | 373 | 0.00 | 373 | 0.54 |
| Zhang Man^61^ | 2006 | Chinese | 2000 | Xinjiang | Cross-sectional Study | Reeducation Center | Convenience Sampling | ELISA/WB | RPR/TPHA | 885 | 124 | 0.81 | 124 | 7.26 |
|  |  |  | 2001 | Xinjiang | Cross-sectional Study | Reeducation Center | Convenience Sampling |  |  |  | 335 | 0.60 | 335 | 7.46 |
|  |  |  | 2002 | Xinjiang | Cross-sectional Study | Reeducation Center | Convenience Sampling |  |  |  | 184 | 1.63 | 184 | 4.35 |
|  |  |  | 2003 | Xinjiang | Cross-sectional Study | Reeducation Center | Convenience Sampling |  |  |  | 134 | 0.75 | 134 | 8.21 |
|  |  |  | 2004 | Xinjiang | Cross-sectional Study | Reeducation Center | Convenience Sampling |  |  |  | 81 | 0.00 | 81 | 3.70 |
| Liu Yingjie^62^ | 2006 | Chinese | 2005 | Beijing | Cross-sectional Study | Reeducation Center | Convenience Sampling | ELISA/WB | RPR/ELISA | 403 | 403 | 0.50 | 403 | 11.66 |
| Shi Xiaoliu^63^ | 2008 | Chinese | 2006 | Sichuan | Cross-sectional Study | Entertainment Venues | Convenience Sampling | ELISA/WB | RPR | 462 | 462 | 0.22 | 462 | 0.22 |
|  |  |  | 2007 | Sichuan | Cross-sectional Study | Entertainment Venues | Convenience Sampling |  |  | 400 | 400 | 0.25 | 400 | 0.00 |
| Li Wenjie^64^ | 2007 | Chinese | 2005 | Guangdong | Cross-sectional Study | Entertainment Venues | Stratified Random Sampling | ELISA/WB | TRUST/ELISA | 285 | 285 | 0.00 | 285 | 14.47 |
| Pan Ganglei^65^ | 2012 | Chinese | 2011 | Zhejiang | Cross-sectional Study | Entertainment Venues | Convenience Sampling | ELISA/WB | RPR/ELISA | 617 | 617 | 0.32 | 617 | 13.29 |
| Cheng Xiaoli^66^ | 2009 | Chinese | 2003 | Anhui | Cross-sectional Study | Reeducation Center | Convenience Sampling | ELISA-1/ELISA-2/WB | TRUST | 368 | 368 | 0.30 | 368 | 16.00 |
|  |  |  | 2004 | Anhui | Cross-sectional Study | Reeducation Center | Convenience Sampling |  |  | 518 | 518 | 0.20 | 518 | 11.00 |
|  |  |  | 2005 | Anhui | Cross-sectional Study | Reeducation Center | Convenience Sampling |  |  | 393 | 393 | 0.80 | 393 | 15.50 |
|  |  |  | 2006 | Anhui | Cross-sectional Study | Reeducation Center | Convenience Sampling |  |  | 401 | 401 | 0.50 | 401 | 12.20 |
|  |  |  | 2007 | Anhui | Cross-sectional Study | Reeducation Center | Convenience Sampling |  |  | 312 | 312 | 0.60 | 312 | 18.30 |
| Liu Cong^67^ | 2011 | Chinese | 2008 | Hubei | Cross-sectional Study | Unspecified | Convenience Sampling | Unspecified | Unspecified | 444 | 444 | 0.00 | 444 | 3.60 |
| Weng Yuqiu^68^ | 2011 | Chinese | 2008 | Guangxi | Cross-sectional Study | Entertainment Venues | Convenience Sampling | ELISA-1/ELISA-2 | RPR | 1047 | 1047 | 0.40 | 1047 | 2.70 |
| Lu Fan^69^ | 2009 | English | 2005 | Guangxi | Cross-sectional Study | Entertainment Venues | RDS||/snowball sampling | ELISA/WB | RPR/TPPA | 362 | 354 | 2.30 | 354 | 11.00 |
| Zhang Yanhui^70^ | 2011 | Chinese | 2008 | Zhejiang | Cross-sectional Study | Entertainment Venues | Convenience Sampling | ELISA/WB | RPR/TPPA | None^¶^ | 460 | 0.00 | 460 | 4.30 |
|  |  |  | 2008 | Chongqing | Cross-sectional Study | Entertainment Venues | Convenience Sampling |  |  |  | 429 | 0.23 | 429 | 1.20 |
|  |  |  | 2008 | Guangdong | Cross-sectional Study | Entertainment Venues | Convenience Sampling |  |  |  | 289 | 0.00 | 289 | 3.50 |
|  |  |  | 2008 | Liaoning | Cross-sectional Study | Entertainment Venues | Convenience Sampling |  |  |  | 601 | 0.00 | 601 | 0.80 |
|  |  |  | 2008 | Shanghai | Cross-sectional Study | Entertainment Venues | Convenience Sampling |  |  |  | 416 | 0.00 | 416 | 2.40 |
|  |  |  | 2008 | Yunnan | Cross-sectional Study | Entertainment Venues | Convenience Sampling |  |  |  | 405 | 0.00 | 405 | 1.50 |
|  |  |  | 2008 | Beijing | Cross-sectional Study | Entertainment Venues | Convenience Sampling |  |  |  | 2688 | 0.00 | 2688 | 1.40 |
|  |  |  | 2008 | Tianjin | Cross-sectional Study | Entertainment Venues | Convenience Sampling |  |  |  | 534 | 0.00 | 534 | 5.90 |
|  |  |  | 2008 | Shanxi | Cross-sectional Study | Entertainment Venues | Convenience Sampling |  |  |  | 400 | 0.00 | 400 | 1.50 |
|  |  |  | 2008 | Hubei | Cross-sectional Study | Entertainment Venues | Convenience Sampling |  |  |  | 444 | 0.00 | 444 | 3.60 |
|  |  |  | 2008 | Hainan | Cross-sectional Study | Entertainment Venues | Convenience Sampling |  |  |  | 400 | 0.25 | 400 | 7.00 |
|  |  |  | 2008 | Hainan | Cross-sectional Study | Entertainment Venues | Convenience Sampling |  |  |  | 404 | 0.00 | 404 | 8.90 |
|  |  |  | 2008 | Heilongj | Cross-sectional Study | Entertainment Venues | Convenience Sampling |  |  |  | 447 | 0.00 | 447 | 3.40 |
|  |  |  | 2008 | Jiangsu | Cross-sectional Study | Entertainment Venues | Convenience Sampling |  |  |  | 400 | 0.00 | 400 | 2.80 |
|  |  |  | 2008 | Shandong | Cross-sectional Study | Entertainment Venues | Convenience Sampling |  |  |  | 400 | 0.00 | 400 | 1.30 |
| Shi Wenya^71^ | 2012 | Chinese | 2006 | Beijing | Cross-sectional Study | Entertainment Venues | Stratified Random Sampling | Unspecified | RPR | 198 | 167 | 0.00 | 167 | 3.00 |
|  |  |  | 2007 | Beijing | Cross-sectional Study | Entertainment Venues | Stratified Random Sampling |  |  | 202 | 192 | 0.00 | 192 | 2.10 |
|  |  |  | 2008 | Beijing | Cross-sectional Study | Entertainment Venues | Stratified Random Sampling |  |  | 208 | 195 | 0.00 | 195 | 1.50 |
|  |  |  | 2009 | Beijing | Cross-sectional Study | Entertainment Venues | Stratified Random Sampling |  |  | 202 | 198 | 0.00 | 198 | 1.00 |
|  |  |  | 2006 | Beijing | Cross-sectional Study | Reeducation Center | Stratified Random Sampling |  |  | 267 | 267 | 0.40 | 267 | 8.60 |
|  |  |  | 2007 | Beijing | Cross-sectional Study | Reeducation Center | Stratified Random Sampling |  |  | 452 | 452 | 0.40 | 452 | 10.40 |
|  |  |  | 2008 | Beijing | Cross-sectional Study | Reeducation Center | Stratified Random Sampling |  |  | 236 | 236 | 0.00 | 236 | 6.40 |
|  |  |  | 2009 | Beijing | Cross-sectional Study | Reeducation Center | Stratified Random Sampling |  |  | 292 | 292 | 0.00 | 292 | 3.80 |
| Dong Xiaoyue^72^ | 2008 | Chinese | 2006 | Tianjin | Cross-sectional Study | Reeducation Center | Convenience Sampling | ELISA/WB | RPR/TPPA | 449 | 449 | 0.45 | 449 | 5.57 |
| Zi Guisheng^73^ | 2009 | Chinese | 2008 | Yunnan | Cross-sectional Study | Entertainment Venues | Convenience Sampling | ELISA/WB | TPHA | 85 | 69 | 5.80 | 69 | 1.45 |
| Zhou Jiahui^74^ | 2010 | Chinese | 2007 | Guangdong | Cross-sectional Study | Entertainment Venues | Convenience Sampling | ELISA/Immunocolloidal gold/WB | TRUST/TPPA | 418 | 352 | 0.00 | 352 | 0.30 |
| Zhou Caixia^75^ | 2011 | Chinese | 2010 | Guizhou | Cross-sectional Study | Entertainment Venues | Convenience Sampling | ELISA-1/ELISA-2 | RPR | 235 | 235 | 0.00 | 235 | 8.50 |
| Zheng Wenai^76^ | 2009 | Chinese | 2002 | Hainan | Cross-sectional Study | Reeducation Center | Convenience Sampling | ELISA | TRUST/TPPA | 1144 | 84 | 0.00 | 84 | 23.81 |
|  |  |  | 2003 | Hainan | Cross-sectional Study | Reeducation Center | Convenience Sampling |  |  |  | 154 | 0.00 | 154 | 36.36 |
|  |  |  | 2004 | Hainan | Cross-sectional Study | Reeducation Center | Convenience Sampling |  |  |  | 173 | 0.00 | 173 | 28.32 |
|  |  |  | 2005 | Hainan | Cross-sectional Study | Reeducation Center | Convenience Sampling |  |  |  | 183 | 0.00 | 183 | 30.05 |
|  |  |  | 2006 | Hainan | Cross-sectional Study | Reeducation Center | Convenience Sampling |  |  |  | 153 | 0.00 | 153 | 26.14 |
|  |  |  | 2007 | Hainan | Cross-sectional Study | Reeducation Center | Convenience Sampling |  |  |  | 103 | 0.00 | 103 | 34.95 |
|  |  |  | 2008 | Hainan | Cross-sectional Study | Reeducation Center | Convenience Sampling |  |  |  | 172 | 0.00 | 172 | 25.00 |
| Zhao Shuhai^77^ | 2007 | Chinese | 2006 | Hunan | Cross-sectional Study | Reeducation Center | Convenience Sampling | ELISA/WB | Immunocolloidal gold | 140 | 79 | 1.27 | 79 | 3.80 |
|  |  |  | 2007 | Hunan | Cross-sectional Study | Reeducation Center | Convenience Sampling |  |  |  | 61 | 0.00 | 61 | 11.48 |
| Zhao Jinzhu^78^ | 2011 | Chinese | 2009 | Hubei | Cross-sectional Study | Entertainment Venues | Convenience Sampling | Unspecified | RPR/TRUST/TPPA | 198 | 163 | 0.00 | 165 | 8.50 |
| Zhang Yingxia^79^ | 2011 | Chinese | 2007 | Guangxi | Cross-sectional Study | Entertainment Venues | Stratified Random Sampling | ELISA-1/ELISA-2 | RPR | 403 | 403 | 0.00 | 403 | 2.48 |
| Zhang Yan^80^ | 2005 | Chinese | 2004 | Shandong | Cross-sectional Study | Entertainment Venues | Convenience Sampling | ELISA | RPR | 199 | 180 | 0.00 | 180 | 0.00 |
| Zhang Qianqian^81^ | 2012 | Chinese | 2009 | Jiangsu | Cross-sectional Study | Entertainment Venues | Convenience Sampling | ELISA/WB | RPR/ELISA | 1806 | 1806 | 0.30 | 1806 | 8.40 |
| Zhang Chunping^82^ | 2008 | Chinese | 2007 | Yunnan | Cross-sectional Study | Entertainment Venues | Convenience Sampling | ELISA | TRUST | 483 | 483 | 0.83 | 483 | 0.83 |
| Yu Kaiwen^83^ | 2010 | Chinese | 2009 | Yunnan | Cross-sectional Study | Entertainment Venues | Convenience Sampling | ELISA-1/ELISA-2 | RPR | 320 | 320 | 1.25 | 320 | 0.83 |
| Yang Junying^84^ | 2011 | Chinese | 2007 | Jiangsu | Cross-sectional Study | Entertainment Venues | Convenience Sampling | ELISA | RPR | 247 | 247 | 0.00 | 247 | 5.26 |
| Yang Beifang^85^ | 2006 | Chinese | 2004 | Hubei | Cross-sectional Study | Entertainment Venues | Convenience Sampling | ELISA/WB | TRUST/TPPA | 937 | 937 | 0.00 | 937 | 2.67 |
| Xu Yongfang^86^ | 2009 | Chinese | 2007 | Guangxi | Cross-sectional Study | Entertainment Venues | Stratified Random Sampling | ELISA-1/ELISA-2 | TRUST/TPPA | 379 | 379 | 0.53 | 379 | 3.69 |
|  |  |  | 2008 | Guangxi | Cross-sectional Study | Entertainment Venues | Stratified Random Sampling |  |  | 400 | 400 | 0.25 | 400 | 6.25 |
| Xu Shiming^87^ | 2006 | Chinese | 2004 | Chongqing | Cross-sectional Study | Reeducation Center | Convenience Sampling | ELISA/WB | RPR/TPHA | 1113 | 1113 | 3.90 | 1113 | 5.50 |
| Xu Huafeng^88^ | 2011 | Chinese | 2009 | Henan | Intervention Study | Entertainment Venues | Convenience Sampling | ELISA/WB | ELISA | 200 | 200 | 0.00 | 200 | 1.00 |
| Xu Jiageng^89^ | 2009 | Chinese | 2008 | Jiangsu | Cross-sectional Study | Unspecified | Convenience Sampling | ELISA | TRUST/TPPA | 52 | 52 | 0.00 | 52 | 3.85 |
| Xia Dongyan^90^ | 2009 | Chinese | 2006 | Beijing | Cross-sectional Study | Entertainment Venues | Stratified Random Sampling | ELISA-1/ELISA-2 | RPR | 2470 | 2419 | 0.12 | 2419 | 1.24 |
|  |  |  | 2007 | Beijing | Cross-sectional Study | Entertainment Venues | Stratified Random Sampling |  |  | 2688 | 2669 | 0.00 | 2669 | 1.39 |
|  |  |  | 2008 | Beijing | Cross-sectional Study | Entertainment Venues | Stratified Random Sampling |  |  | 2640 | 2607 | 0.04 | 2607 | 0.77 |
| Wu Chunlin^91^ | 2010 | Chinese | 2007 | Sichuan | Cross-sectional Study | Entertainment Venues | Stratified Random Sampling | Unspecified | Unspecified | 444 | 444 | 0.68 | 444 | 7.90 |
| Wei Zhengya^92^ | 2010 | Chinese | 2009 | Jiangsu | Cross-sectional Study | Entertainment Venues | Convenience Sampling | ELISA | RPR/ELISA | 940 | 940 | 0.00 | 940 | 8.40 |
| Wei Qihou^93^ | 2008 | Chinese | 2005 | Guangxi | Cross-sectional Study | Entertainment Venues and Reeducation Center | Convenience Sampling | Unspecified | TRUST | 392 | 392 | 1.30 | 392 | 15.10 |
| Wang Wenming^94^ | 2008 | Chinese | 2007 | Jiangsu | Cross-sectional Study | Reeducation Center | Convenience Sampling | ELISA | RPR/ELISA | 297 | 297 | 0.00 | 297 | 9.09 |
| Wang Wanwei^95^ | 2010 | Chinese | 2009 | Sichuan | Cross-sectional Study | Entertainment Venues | Stratified Random Sampling | ELISA-1/ELISA-2 | RPR | 1160 | 1156 | 0.43 | 1156 | 0.69 |
| Wang Jinghua^96^ | 2010 | Chinese | 2009 | Shandong | Cross-sectional Study | Entertainment Venues | Convenience Sampling | ELISA-1/ELISA-2/WB | TRUST/TPPA | 348 | 348 | 0.86 | 348 | 2.30 |
| Wang Jinyu^97^ | 2010 | Chinese | 2009 | Guangdong | Cross-sectional Study | Entertainment Venues | Convenience Sampling | ELISA/WB | RPR | 543 | 543 | 0.18 | 543 | 0.92 |
| Wang Guixiang^98^ | 2008 | Chinese | 2006 | Yunnan | Cross-sectional Study | Entertainment Venues | Convenience Sampling | ELISA/WB | RPR/TPPA | 753 | 747 | 11.91 | 747 | 8.43 |
| Wang Bingfa^99^ | 2004 | Chinese | 2002 | Fujian | Cross-sectional Study | Reeducation Center | Convenience Sampling | ELISA/WB | TRUST/TPPA | 267 | 267 | 0.00 | 267 | 20.60 |
| Wang Biao^100^ | 2010 | Chinese | 2009 | Jiangsu | Cross-sectional Study | Entertainment Venues | Convenience Sampling | ELISA/WB | RPR/TPPA | 453 | 400 | 0.00 | 400 | 4.00 |
| Wang Haibo^101^ | 2007 | Chinese | 2006 | Yunnan | Cross-sectional Study | Entertainment Venues | Convenience Sampling | ELISA/WB | RPR/TPPA | 837 | 837 | 8.20 | 837 | 10.10 |
| Wang Fanghua^102^ | 2009 | Chinese | 2008 | Anhui | Cross-sectional Study | Entertainment Venues | Convenience Sampling | ELISA-1/ELISA-2/WB | ELISA | 4584 | 4584 | 0.02 | 4584 | 2.29 |
| Tao Liandi^103^ | 2011 | Chinese | 2010 | Gansu | Cross-sectional Study | Entertainment Venues | Stratified Random Sampling | ELISA/WB | USR | 1200 | 1200 | 0.00 | 1200 | 9.00 |
| Tang Yaqing^104^ | 2007 | Chinese | 2006 | Beijing | Cross-sectional Study | Entertainment Venues | Convenience Sampling | ELISA/WB | ELISA | 105 | 105 | 0.00 | 105 | 0.95 |
| Tang Mengjin^105^ | 2009 | Chinese | 2007 | Guangxi | Cross-sectional Study | Entertainment Venues | Convenience Sampling | ELISA-1/ELISA-2 | RPR | 362 | 362 | 1.10 | 362 | 2.49 |
| Tan Yong^106^ | 2011 | Chinese | 2006 | Sichuan | Cross-sectional Study | Entertainment Venues | Convenience Sampling | ELISA-1/ELISA-2 | RPR | 406 | 328 | 0.60 | 326 | 2.50 |
|  |  |  | 2010 | Sichuan | Cross-sectional Study | Entertainment Venues | Convenience Sampling |  |  | 400 | 328 | 0.00 | 399 | 6.50 |
| Tan Shounan^107^ | 2010 | Chinese | 2007 | Guangxi | Cross-sectional Study | Entertainment Venues | Convenience Sampling | Unspecified | Unspecified | 246 | 246 | 2.03 | 246 | 1.22 |
| Tan Jingguang^108^ | 2009 | Chinese | 2008 | Guangdong | Cross-sectional Study | Entertainment Venues | Stratified Random Sampling | Unspecified | Unspecified | 335 | 335 | 0.00 | 335 | 2.40 |
| Tan Weiwei^109^ | 2008 | Chinese | 2007 | Guangxi | Cross-sectional Study | Entertainment Venues | Convenience Sampling | ELISA/WB | RPR/TPPA | 400 | 379 | 0.50 | 379 | 3.20 |
| Suo Jianming^110^ | 2011 | Chinese | 2010 | Shanxi | Cross-sectional Study | Entertainment Venues | Convenience Sampling | Immunocolloidal gold/WB | TPHA | 96 | 96 | 0.00 | 96 | 1.04 |
| Sun Zhixia^111^ | 2008 | Chinese | 2006 | Zhejiang | Cross-sectional Study | Reeducation Center | Convenience Sampling | ELISA/WB | RPR/TPPA | 141 | 141 | 0.00 | 141 | 7.09 |
| Song Shujuan^112^ | 2008 | Chinese | 2006 | Zhejiang | Cross-sectional Study | Reeducation Center | Convenience Sampling | ELISA/WB | RPR/TPPA | 272 | 272 | 0.00 | 272 | 25.65 |
| Shi Wenya^113^ | 2007 | Chinese | 2005 | Beijing | Cross-sectional Study | Unspecified | Convenience Sampling | ELISA | ELISA | 114 | 109 | 0.00 | 109 | 5.50 |
| Ren Xianyun^114^ | 2006 | Chinese | 2004 | Inner Mongolia | Cross-sectional Study | Entertainment Venues | Convenience Sampling | ELISA/WB | ELISA | 463 | 463 | 0.00 | 463 | 4.50 |
| Qiu Jinjun^115^ | 2008 | Chinese | 2007 | Guangdong | Cross-sectional Study | Entertainment Venues | Convenience Sampling | ELISA-1/ELISA-2 | RPR | 413 | 413 | 0.24 | 413 | 4.40 |
| Qin Yanmin^116^ | 2010 | Chinese | 2007 | Guangdong | Cross-sectional Study | Reeducation Center | Convenience Sampling | ELISA/WB | RPR/TPPA | 262 | 262 | 0.00 | 262 | 7.63 |
|  |  |  | 2008 | Guangdong | Cross-sectional Study | Reeducation Center | Convenience Sampling |  |  | 311 | 311 | 0.00 | 311 | 5.14 |
| Qi Hezhen^117^ | 2007 | Chinese | 2005 | Jiangsu | Cross-sectional Study | Reeducation Center | Convenience Sampling | ELISA | RPR/TPPA | 207 | 207 | 0.00 | 207 | 5.31 |
| Pan Xinlian^118^ | 2008 | Chinese | 2006 | Guangxi | Cross-sectional Study | Entertainment Venues | Convenience Sampling | ELISA-1/ELISA-2 | RPR | 253 | 253 | 0.79 | 253 | 4.35 |
| Pan Xinlian^119^ | 2009 | Chinese | 2007 | Guangxi | Cross-sectional Study | Entertainment Venues | Convenience Sampling | ELISA-1/ELISA-2 | RPR | 300 | 300 | 1.00 | 300 | 5.67 |
|  |  |  | 2008 | Guangxi | Cross-sectional Study | Entertainment Venues | Convenience Sampling |  |  | 311 | 311 | 0.64 | 311 | 8.68 |
| Ouyang Ning^120^ | 2010 | Chinese | 2009 | Yunnan | Cross-sectional Study | Entertainment Venues | Convenience Sampling | Unspecified | Unspecified | 372 | 372 | 1.60 | 372 | 0.50 |
| Ni Mingjian^121^ | 2005 | Chinese | 2004 | Xinjiang | Cross-sectional Study | Entertainment Venues | Stratified Random Sampling | ELISA-1/ELISA-2 | RPR | 300 | 300 | 0.67 | 300 | 8.00 |
| Mei Lin^122^ | 2009 | Chinese | 2007 | Shanxi | Cross-sectional Study | Entertainment Venues | Convenience Sampling | ELISA/WB | RPR/TPPA | 483 | 481 | 0.00 | 481 | 3.53 |
| Luo Yan^123^ | 2008 | Chinese | 2006 | Zhejiang | Cross-sectional Study | Reeducation Center | Convenience Sampling | ELISA-1/ELISA-2 | RPR | 262 | 262 | 0.00 | 262 | 17.60 |
| Luo Jie^124^ | 2005 | Chinese | 2004 | Guangxi | Cross-sectional Study | Entertainment Venues and Reeducation Center | Convenience Sampling | ELISA-1/ELISA-2 | RPR | 362 | 362 | 3.00 | 362 | 11.00 |
| Lu Hua^125^ | 2003 | Chinese | 2002 | Xinjiang | Cross-sectional Study | Reeducation Center | Convenience Sampling | ELISA-1/ELISA-2 | RPR | 346 | 346 | 0.29 | 346 | 7.23 |
| Liu Xuezhen^126^ | 2006 | Chinese | 2004 | Shandong | Cross-sectional Study | Unspecified | Convenience Sampling | ELISA-1/ELISA-2 | RPR | 3513 | 3513 | 0.03 | 3513 | 2.19 |
| Liu Wangbo^127^ | 2010 | Chinese | 2007 | Jilin | Cross-sectional Study | Entertainment Venues | Stratified Random Sampling | ELISA-1/ELISA-2 | RPR | 410 | 410 | 0.00 | 410 | 1.00 |
| Liu Lirong^128^ | 2007 | Chinese | 2006 | Beijing | Cross-sectional Study | Entertainment Venues | Convenience Sampling | ELISA-1/ELISA-2 | RPR | 341 | 341 | 0.00 | 341 | 1.17 |
| Liu Caiqiong^129^ | 2010 | Chinese | 2007 | Yunnan | Intervention Study | Entertainment Venues | Convenience Sampling | Unspecified | Unspecified | 365 | 365 | 1.64 | 365 | 4.11 |
| Liao Meizhen^130^ | 2008 | Chinese | 2007 | Shandong | Cross-sectional Study | Entertainment Venues | Convenience Sampling | ELISA-1/ELISA-2 | RPR/TPHA | 6811 | 6705 | 0.01 | 6488 | 1.46 |
| Liang Shuping^131^ | 2010 | Chinese | 2008 | Ningxia | Intervention Study | Entertainment Venues | Convenience Sampling | Unspecified | Unspecified | 307 | 307 | 0.00 | 307 | 14.00 |
| Li Xiaoyu^132^ | 2011 | Chinese | 2010 | Zhejiang | Cross-sectional Study | Reeducation Center | Convenience Sampling | ELISA | RPR/TPPA | 98 | 98 | 0.00 | 98 | 25.51 |
| Li Yanfen^133^ | 2011 | Chinese | 2010 | Shanghai | Cross-sectional Study | Entertainment Venues | Convenience Sampling | ELISA | RPR/ELISA | 370 | 370 | 0.00 | 370 | 3.50 |
| Li Yan^134^ | 2009 | Chinese | 2006 | Guangdong | Cross-sectional Study | Entertainment Venues | RDS||/snowball sampling | ELISA/WB | RPR/TPHA | 320 | 320 | 0.00 | 320 | 8.00 |
| Li Xiaojing^135^ | 2006 | Chinese | 2002 | Anhui | Cross-sectional Study | Entertainment Venues | Convenience Sampling | ELISA-1/ELISA-2 | RPR | 403 | 403 | 0.20 | 403 | 23.30 |
|  |  |  | 2003 | Anhui | Cross-sectional Study | Entertainment Venues | Convenience Sampling |  |  | 368 | 368 | 0.30 | 368 | 16.00 |
|  |  |  | 2004 | Anhui | Cross-sectional Study | Entertainment Venues | Convenience Sampling |  |  | 518 | 518 | 0.20 | 518 | 11.00 |
| Li Wenzheng^136^ | 2009 | Chinese | 2008 | Yunnan | Cross-sectional Study | Entertainment Venues | Stratified Random Sampling | ELISA/WB | RPR/TPHA | 405 | 405 | 2.20 | 405 | 1.50 |
| Li Jinxing^137^ | 2007 | Chinese | 2003 | Shandong | Cross-sectional Study | Entertainment Venues | Convenience Sampling | ELISA-1/ELISA-2/WB | TRUST | 500 | 500 | 0.00 | 500 | 0.60 |
|  |  |  | 2004 | Shandong | Cross-sectional Study | Entertainment Venues | Convenience Sampling |  |  | 640 | 640 | 0.16 | 640 | 0.63 |
|  |  |  | 2005 | Shandong | Cross-sectional Study | Entertainment Venues | Convenience Sampling |  |  | 400 | 400 | 0.25 | 400 | 1.25 |
| Li Fen^138^ | 2012 | Chinese | 2011 | Beijing | Cross-sectional Study | Entertainment Venues | Stratified Random Sampling | ELISA/WB | RPR | 290 | 290 | 0.00 | 290 | 1.70 |
| Lei Zhangquan^139^ | 2005 | Chinese | 2004 | Sichuan | Cross-sectional Study | Entertainment Venues | Convenience Sampling | ELISA/WB | TRUST/TPHA | 259 | 243 | 3.29 | 243 | 6.17 |
| Jin Yuelong^140^ | 2009 | Chinese | 2008 | Anhui | Cross-sectional Study | Entertainment Venues | Stratified Random Sampling | ELISA | RPR | 500 | 500 | 0.00 | 500 | 2.75 |
| Ji Chunhua^141^ | 2009 | Chinese | 2004 | Shanxi | Cross-sectional Study | Entertainment Venues | Convenience Sampling | ELISA | RPR/TPHA | 296 | 296 | 0.00 | 296 | 0.68 |
|  |  |  | 2005 | Shanxi | Cross-sectional Study | Entertainment Venues | Convenience Sampling |  |  | 305 | 305 | 0.00 | 305 | 0.00 |
|  |  |  | 2006 | Shanxi | Cross-sectional Study | Entertainment Venues | Convenience Sampling |  |  | 303 | 303 | 0.00 | 303 | 0.00 |
| Huang Kezan^142^ | 2010 | Chinese | 2009 | Guangdong | Cross-sectional Study | Entertainment Venues | Stratified Random Sampling | ELISA/WB | TRUST/ELISA | 391 | 391 | 0.77 | 391 | 2.81 |
| Huang Jinfei^143^ | 2010 | Chinese | 2009 | Guangdong | Cross-sectional Study | Entertainment Venues | Convenience Sampling | ELISA/WB | ELISA | 333 | 333 | 0.00 | 333 | 7.51 |
| Huang Guanglan^144^ | 2012 | Chinese | 2011 | Guangxi | Cross-sectional Study | Entertainment Venues | Stratified Random Sampling | ELISA | RPR | 301 | 301 | 0.66 | 301 | 4.32 |
| Hu Xiaoqian^145^ | 2011 | Chinese | 2010 | Shaanxi | Cross-sectional Study | Entertainment Venues | Convenience Sampling | ELISA-1/ELISA-2/WB | RPR/ELISA | 400 | 400 | 0.00 | 400 | 2.75 |
| Gao Linlin^146^ | 2008 | Chinese | 2007 | Yunnan | Cross-sectional Study | Reeducation Center | Convenience Sampling | ELISA/WB | TRUST/TPPA | 270 | 270 | 3.30 | 270 | 11.90 |
| Gao Jianmei^147^ | 2008 | Chinese | 2006 | Beijing | Cross-sectional Study | Entertainment Venues | Stratified Random Sampling | ELISA/WB | RPR/ELISA | 102 | 102 | 0.00 | 102 | 4.90 |
| Feng Ning^148^ | 2011 | Chinese | 2010 | Shanxi | Cross-sectional Study | Entertainment Venues | Stratified Random Sampling | ELISA/WB | TRUST/ELISA/TPPA | 400 | 400 | 0.00 | 400 | 1.50 |
| Du Jiaquan^149^ | 2008 | Chinese | 2006 | Yunnan | Cross-sectional Study | Entertainment Venues | Convenience Sampling | Unspecified | Unspecified | 366 | 363 | 16.25 | 363 | 11.02 |
| Dong Xiaoyue^150^ | 2009 | Chinese | 2007 | Tianjin | Cross-sectional Study | Reeducation Center | Convenience Sampling | ELISA-1/ELISA-2 | RPR/TPPA | 178 | 178 | 0.00 | 178 | 11.24 |
| Ding Xianbin^151^ | 2006 | Chinese | 2005 | Chongqing | Cross-sectional Study | Entertainment Venues | Convenience Sampling | ELISA/WB | RPR/TPHA | 519 | 519 | 0.40 | 519 | 2.10 |
| Dai Xiangnong^152^ | 2009 | Chinese | 2006 | Guangdong | Cross-sectional Study | Reeducation Center | Convenience Sampling | ELISA/WB | RPR/TPPA | 202 | 202 | 0.50 | 202 | 14.90 |
| Cui Yuzhe^153^ | 2011 | Chinese | 2006 | Heilongj | Cross-sectional Study | Entertainment Venues | Stratified Random Sampling | ELISA/WB | RPR/TPPA | 417 | 417 | 0.00 | 417 | 1.20 |
|  |  |  | 2007 | Heilongj | Cross-sectional Study | Entertainment Venues | Stratified Random Sampling |  |  | 447 | 447 | 0.00 | 447 | 3.40 |
|  |  |  | 2008 | Heilongj | Cross-sectional Study | Entertainment Venues | Stratified Random Sampling |  |  | 410 | 410 | 0.00 | 410 | 2.40 |
|  |  |  | 2009 | Heilongj | Cross-sectional Study | Entertainment Venues | Stratified Random Sampling |  |  | 447 | 447 | 0.00 | 447 | 2.50 |
| Chen Yilin^154^ | 2009 | Chinese | 2001 | Fujian | Cross-sectional Study | Reeducation Center | Convenience Sampling | ELISA/WB | TRUST/TPPA | 267 | 267 | 0.00 | 267 | 6.00 |
|  |  |  | 2002 | Fujian | Cross-sectional Study | Reeducation Center | Convenience Sampling |  |  | 267 | 267 | 0.00 | 267 | 6.00 |
|  |  |  | 2003 | Fujian | Cross-sectional Study | Reeducation Center | Convenience Sampling |  |  | 252 | 252 | 0.00 | 252 | 16.27 |
|  |  |  | 2004 | Fujian | Cross-sectional Study | Reeducation Center | Convenience Sampling |  |  | 254 | 254 | 0.00 | 254 | 16.93 |
|  |  |  | 2005 | Fujian | Cross-sectional Study | Reeducation Center | Convenience Sampling |  |  | 345 | 345 | 0.00 | 345 | 7.83 |
|  |  |  | 2006 | Fujian | Cross-sectional Study | Reeducation Center | Convenience Sampling |  |  | 251 | 251 | 0.40 | 251 | 9.56 |
|  |  |  | 2007 | Fujian | Cross-sectional Study | Reeducation Center | Convenience Sampling |  |  | 223 | 223 | 0.00 | 223 | 11.66 |
| Chen Wei^155^ | 2011 | Chinese | 2009 | Guangxi | Cross-sectional Study | Entertainment Venues | Stratified Random Sampling | ELISA/WB | RPR | 400 | 400 | 0.25 | 400 | 2.50 |
| Chen Shuxia^156^ | 2011 | Chinese | 2009 | Shandong | Cross-sectional Study | Entertainment Venues | Stratified Random Sampling | ELISA-1/ELISA-2/WB | TRUST/TPPA | 236 | 236 | 0.00 | 236 | 5.51 |
| Chen Shiping^157^ | 2010 | Chinese | 2007 | Jiangxi | Cross-sectional Study | Entertainment Venues | Convenience Sampling | ELISA/WB | RPR | 360 | 149 | 0.00 | 149 | 0.00 |
| Chen Meifen^158^ | 2011 | Chinese | 2010 | Zhejiang | Cross-sectional Study | Entertainment Venues | Convenience Sampling | ELISA/WB | TRUST/TPPA | 739 | 739 | 0.27 | 739 | 0.95 |
| Chen Lin^159^ | 2010 | Chinese | 2009 | Guangdong | Cross-sectional Study | Entertainment Venues | Convenience Sampling | Unspecified | Unspecified | 426 | 426 | 0.00 | 426 | 4.50 |
| Chen Lin^160^ | 2009 | Chinese | 2007 | Guangdong | Cross-sectional Study | Entertainment Venues | Convenience Sampling | Unspecified | Unspecified | 413 | 413 | 0.24 | 413 | 4.40 |
| Chen Lin^161^ | 2006 | Chinese | 2004 | Guangdong | Cross-sectional Study | Reeducation Center | Convenience Sampling | ELISA | Unspecified | 245 | 245 | 0.00 | 245 | 10.00 |
| Chen Fachun^162^ | 2011 | Chinese | 2010 | Hubei | Cross-sectional Study | Entertainment Venues | Stratified Random Sampling | ELISA-1/ELISA-2 | TRUST/ELISA | 400 | 400 | 0.00 | 400 | 2.50 |
| Cao Xiaoyun^163^ | 2006 | Chinese | 2005 | Sichuan | Cross-sectional Study | Entertainment Venues | Convenience Sampling | ELISA/WB | ELISA | 343 | 343 | 0.60 | 343 | 18.10 |
| Cao Jianxin^164^ | 2008 | Chinese | 2004 | Shanxi | Cross-sectional Study | Entertainment Venues | Convenience Sampling | ELISA-1/ELISA-2/WB | RPR | 339 | 339 | 0.00 | 339 | 2.95 |
|  |  |  | 2005 | Shanxi | Cross-sectional Study | Entertainment Venues | Convenience Sampling |  |  | 238 | 238 | 0.00 | 238 | 0.00 |
|  |  |  | 2006 | Shanxi | Cross-sectional Study | Entertainment Venues | Convenience Sampling |  |  | 288 | 288 | 0.00 | 288 | 1.39 |
| Cao Hui^165^ | 2010 | Chinese | 2010 | Tianjin | Cross-sectional Study | Entertainment Venues | Convenience Sampling | Unspecified | Unspecified | 186 | 186 | 0.00 | 186 | 7.00 |
| Cai Ying^166^ | 2010 | Chinese | 2005 | Yunnan | Cross-sectional Study | Entertainment Venues | Convenience Sampling | ELISA/WB | Unspecified | 418 | 387 | 1.55 | 387 | 4.13 |
|  |  |  | 2009 | Yunnan | Cross-sectional Study | Entertainment Venues | Convenience Sampling |  |  | 410 | 408 | 0.49 | 408 | 1.47 |
| Bo Fubao^167^ | 2007 | Chinese | 2006 | Inner Mongolia | Cross-sectional Study | Entertainment Venues | Stratified Random Sampling | ELISA-1/ELISA-2 | ELISA | 624 | 624 | 0.00 | 624 | 9.46 |
| Bai Yu^168^ | 2010 | Chinese | 2008 | Guangxi | Cross-sectional Study | Entertainment Venues | Convenience Sampling | ELISA-1/ELISA-2 | RPR/TPPA | 1047 | 1047 | 0.40 | 1047 | 2.70 |
| Bai Junmei^169^ | 2006 | Chinese | 2006 | Beijing | Cross-sectional Study | Entertainment Venues | Stratified Random Sampling | ELISA/WB | RPR/ELISA | 114 | 109 | 0.00 | 109 | 5.50 |
| Bang-Yang Zhu^170^ | 2012 | English | 2007 | Guangxi | Cross-sectional Study | Entertainment Venues | Convenience Sampling | ELISA-1/ELISA-2/WB | TRUST/TPPA | 488 | 488 | 0.80 | 488 | 7.17 |
| JUN JIE XU^171^ | 2008 | English | 2006 | Yunnan | Cross-sectional Study | Entertainment Venues | Convenience Sampling | ELISA/WB | RPR/TPPA | 98 | 98 | 8.30 | 98 | 12.50 |
| YUHUA RUAN^172^ | 2006 | English | 2005 | Sichuan | Cross-sectional Study | Entertainment Venues | Convenience Sampling | ELISA/WB | ELISA/TPPA | 343 | 343 | 0.60 | 343 | 15.70 |
| Meizhen Liao^173^ | 2012 | English | 2008 | Shandong | Cross-sectional Study | Entertainment Venues | RDS||/snowball sampling | ELISA-1/ELISA-2/WB | RPR/TPPA | 363 | 363 | 0.00 | 363 | 2.80 |
|  |  |  | 2009 | Shandong | Cross-sectional Study | Entertainment Venues | RDS||/snowball sampling |  |  | 432 | 432 | 0.00 | 432 | 2.20 |
| Xiangsheng Chen^174^ | 2005 | English | 2000 | Yunnan | Cross-sectional Study | Reeducation Center and STD Clinic | Convenience Sampling | ELISA/WB | RPR/TPHA | 505 | 505 | 10.30 | 505 | 9.50 |
| Zhao Yanqiu^175^ | 2010 | Chinese | 2008 | Jiangsu | Cross-sectional Study | Entertainment Venues | Stratified Random Sampling | ELISA/WB | RPR/TPPA | 400 | 396 | 0.30 | 396 | 2.00 |
| Wei Xingwu^176^ | 2002 | Chinese | 2001 | Xinjiang | Cross-sectional Study | Entertainment Venues | Stratified Random Sampling | Unspecified | Unspecified | 181 | 181 | 0.00 | 181 | 3.31 |
| Qi Hezhen^177^ | 2003 | Chinese | 2000 | Jiangsu | Cross-sectional Study | Reeducation Center | Convenience Sampling | ELISA/WB | RPR/TPPA | 153 | 153 | 0.00 | 153 | 9.15 |
|  |  |  | 2001 | Jiangsu | Cross-sectional Study | Reeducation Center | Convenience Sampling |  |  | 177 | 177 | 0.00 | 177 | 14.12 |
| Ye Yongqing^178^ | 2005 | Chinese | 2003 | Xinjiang | Cross-sectional Study | Entertainment Venues | Convenience Sampling | Unspecified | Unspecified | 459 | 459 | 0.00 | 459 | 0.87 |
| Xue Fanghui^179^ | 2006 | Chinese | 2005 | Henan | Cross-sectional Study | Entertainment Venues | Stratified Random Sampling | ELISA/WB | RPR/TPPA | 207 | 207 | 0.00 | 207 | 2.42 |
| Luo Zhen^180^ | 2007 | Chinese | 2006 | Shanghai | Cross-sectional Study | Entertainment Venues and Reeducation Center | Convenience Sampling | Unspecified | RPR | 529 | 373 | 0.00 | 373 | 4.83 |
| Ao Xiu^181^ | 2008 | Chinese | 2006 | Beijing | Cross-sectional Study | Entertainment Venues | Stratified Random Sampling | ELISA-1/ELISA-2/WB | RPR/ELISA | 105 | 105 | 0.95 | 105 | 2.86 |
| Huang Yunkun^182^ | 2008 | Chinese | 2006 | Guangxi | Intervention Study | Entertainment Venues | Stratified Random Sampling | Unspecified | Unspecified | 380 | 380 | 0.79 | 380 | 5.53 |
| Luo Xiaoying^183^ | 2010 | Chinese | 2009 | Zhejiang | Cross-sectional Study | Entertainment Venues | Stratified Random Sampling | ELISA | TRUST/TPPA | 530 | 530 | 0.19 | 530 | 2.45 |
| Zhang Guisong^184^ | 2009 | Chinese | 2005 | Guangdong | Cross-sectional Study | Entertainment Venues | Convenience Sampling | ELISA/WB | ELISA | 512 | 325 | 0.00 | 325 | 4.00 |
| Yin Fanglan^185^ | 2009 | Chinese | 2008 | Shanghai | Cross-sectional Study | Entertainment Venues | Stratified Random Sampling | ELISA-1/ELISA-2 | RPR | 452 | 452 | 0.00 | 452 | 3.30 |
| Luo Lei^186^ | 2009 | Chinese | 2007 | Sichuan | Cross-sectional Study | Entertainment Venues | Convenience Sampling | ELISA-1/ELISA-2 | RPR | 411 | 410 | 2.49 | 410 | 5.85 |
| Chen Jiqing^187^ | 2009 | Chinese | 2008 | Zhejiang | Cross-sectional Study | STD Clinic | Convenience Sampling | Unspecified | Unspecified | 168 | 168 | 1.19 | 168 | 9.52 |
| Xia Guomin^188^ | 2008 | Chinese | 2006 | Anhui | Cross-sectional Study | Entertainment Venues | Stratified Random Sampling | ELISA | RPR | 423 | 400 | 0.00 | 400 | 0.25 |
| Luo Xiaorong^189^ | 2008 | Chinese | 2006 | Sichuan | Cross-sectional Study | Entertainment Venues | Stratified Random Sampling | ELISA/WB | RPR/TPPA | 216 | 216 | 0.00 | 216 | 6.94 |
| Chen Yuhong^190^ | 2012 | Chinese | 2011 | Jiangsu | Cross-sectional Study | Entertainment Venues | Convenience Sampling | ELISA | TRUST/ELISA | 400 | 400 | 0.00 | 400 | 6.00 |

^*^Includes four three-year periods: 2000–2002, 2003–2005, 2006–2008, 2009­–2011.

^†^Categorized into seven groups by geographical location: North China (Beijing, Tianjin, Hebei, Shanxi and Inner Mongolia), Northeast (Helongjiang, Jilin and Liaoning), East China (Shanghai, Jiangsu, Zhejiang, Anhui, Fujian, Jiangxi and Shandong), Central China (Henan, Hubei and Hunan), South China (Guangdong, Guangxi and Hainan), Southwest (Chongqing, Sichuan, Guizhou and Yunnan) and Northwest (Shaanxi, Gansu, Ningxia and Xinjiang).

^‡^Categorized into Entertainment Venues, Reeducation Centers and Others (Unspecified, Entertainment Venues and Reeducation Centers, Medical Institutions, Reeducation Centers and STD Clinic and STD Clinic).

^§^Sorted into three groups: <200, 200–400 and ≥400.

^||^RDS: Respondent Driven Sampling.

^¶^No specified sample size.

^**^Sorted into three groups: Confirmatory tests (ELISA + Western blot, ELISA-1 + ELISA-2, ELISA-1 + ELISA-2 + Western blot, Immunocolloidal gold + Western blot, ELISA + Immunocolloidal gold + Western blot), Single method (single ELISA), and Unspecified (exact methods not presented although diagnosis of HIV infection based on positive serological tests).

^††^Sorted into three groups: Treponemal tests (*Treponema pallidum* particle agglutination assay – TPPA, Enzyme-Linked Immuno Sorbent Assay – ELISA, *Treponema pallidum* hemagglutination assay – TPHA, Immunocolloidal gold), Nontreponemal tests (Rapid plasma reagin – RPR, Toluidine red unheated serum test – TRUST, Unheated serum reagin-USR), and Unspecified (exact methods not presented although diagnosis of syphilis infection based on positive serological tests).

^‡‡^References:

1. Wen XQ (2009) SURVEY ON KAB AND SEROLOGY OF AIDS AMONG 360 FEMALE COMMERCIAL SEX WORKERS IN GUILIN. Modern Preventive Medicine 36: 2687-2689.
2. Li DM, Yuan F, Hu SY, Lu F (2007) High risk behaviors and HIV/STI prevalence among female sex workers in different settings. Chin J AIDS STD 13.
3. Hong H, Xu GZ, Zhang DD (2010) Long-term follow-up of a comprehensive HIV and sexually transmitted infection prevention program for female sex workers in Ningbo, China. Int J Gynaecol Obstet 111: 180-181.
4. Xu YJ, Wang SP, Xue ZD, Shen JP (2011) AIDS knowledge and high-risk behavior survey among FSWs in Shanxi Province in 2009. Chinese Remedies and Clinics 11: 304-306.
5. Nie ZQ, Ling P, Li Y, Wang Y (2011) Surveillance of AIDS high-risk people in Guangdong province, 2009. J Trop Med 11: 29-31+45.
6. Zhong J, Lin J, Hu YM, Tan LL, Wang G (2011) HIV/STD infection and risk behaviors among commercial sex workers at various places in Wuzhou City, Guangxi Zhuang Autonomous Rgion. Chinese Journal of Health Education 27: 177-180.
7. Guo YY, Zhang GX, Wang HQ (2010) Analysis of HIV, syphilis and gonococcus infection rate among FSWs in Xiaoshan District in Hangzhou City between 2001 and 2007. Chinese Journal of Rural Medicine and Pharmacy 17.
8. Han WX, Pu YC, Duan QX, Yin LX, Zhang HM, et al. (2012) Comprehensive AIDS surveillance among female sex workers in Longchuan county from 2007 to 2009. Chinese Rural Health Service Administration 32.
9. Yang MX, Cai XF, Li SS (2009) [Intervention modes and cross-sectional survey on AIDS behavior among female sex workers in entertaining establishing in Shanghai]. Zhonghua Liu Xing Bing Xue Za Zhi 30: 419-420.
10. Wang YX, Kang DM, Liao MZ, Tao XR, Wang GY, et al. (2011) [Analysis of condom use and its factors on female sex workers in Shandong province]. Zhonghua Yu Fang Yi Xue Za Zhi 45: 435-439.
11. Wang L, Ding ZW, Ding GW, Guo W, Qin QQ, et al. (2009) Data analysis of national HIV comprehensive surveillance sites among female sex workers from 2004 to 2008. Zhonghua Yu Fang Yi Xue Za Zhi 43: 1009-1015.
12. Tang ZL, Li XF, Dong XP, Wang YF, Chen GZ (2011) Infection of HIV/STDs through sexual behavior among female sex workers during menstruation. Chin J Public Health 27: 1510-1512.
13. Li MQ (2010) Sentinel Surveillance of AIDS in Liuzhou, 2008. J Prev Med Inf Ma 26.
14. Peng H, Yang LG, Zhang MM, Wang H, Huang XX, et al. (2008) AIDS/STD knowledge, attitude and behavior survey among 60 street-based FSWs. Chin J AIDS STD 14: 628-629.
15. Yang P, Wang QQ, Peng H, He L (2009) A survey of syphilis and HIV infection in medium-low-income female sex workers. China J Lepr Skin Dis 25: 174-176.
16. Ouyang H (2012) AIDS related knowledge and STD infection survey among FSWs in Hejiang County in 2011. Journal of Occupational Health and Damage 27: 126-127.
17. Pei DN, Yang B, Lai SZ, Wang FQ (2002) High risk behavior investigation and syphilis survelliance among female sex workers and drug users in Hainan. Chin J STD/AIDS Prev Cont 8: 160-161.
18. Wang HB, Smith K, Brown KS, Wang GX, Chang DF, et al. (2011) Prevalence, incidence, and persistence of syphilis infection in female sex workers in a Chinese province. Epidemiol Infect 139: 1401-1409.
19. Xu JJ, Brown K, Ding G, Wang H, Zhang G, et al. (2011) Factors associated with HIV testing history and HIV-test result follow-up among female sex workers in two cities in Yunnan, China. Sex Transm Dis 38: 89-95.
20. Luo Y, Chen SC, Xu K, Yuan H, Cheng J, et al. (2008) Survey of STD/AIDS-related knowledge, behaviors and infection rates of sex workers in entertainment places in Hangzhou. Disease Surveillance 23: 607-609.
21. Jiang YJ, Shang H, Wang YN, Zhao M, Cao JJ, et al. (2002) Survey of HIV, HBV, HCV and syphilis infection among risk groups in Shenyang. Chin J AIDS/STD Prev cont 8.
22. Miao XF, Zhao HR, Li QM, Li JJ, Zong XM, et al. (2009) The Character of Commercial Sex Workers and HIV/Syphilis/HBsAg Infection in Low - grade P lace of Rural Areas. Chin J Pest Control 25: 813-815.
23. Jiang N (2012) Surveillance of risk behawiors facilitating among commercial sex works and analysis of HIV, Syphilis, HCV and HBV infection. J Medical Forum 33.
24. Chen GS, Wu HS, Yao ZM (2010) A survey of AIDS infection rate and AIDS related knowledge among commercial sex workers in a county in Jiangxi Province. Anhui J Prev Med 16.
25. Zhao GD, Zhong L, Li YY (2011) Analysis of AIDS surveillance results among FSWs in Shangluo City in 2010. J North China Coal Medical University 13: 469-470.
26. Zhang Q, Wang Y, Li P (2008) Study of HIV /TP/HCV infection of 394 prostitutes in Wuhu City. Chinese Journal of Health Laboratory Technology 18: 1865-1868.
27. Zhang MN, Zhang ZH, Huang L, Wang XM (2011) Analysis of the STD and behavior survey among FSWs in a city in Shanxi Province. Chinese Remedies and Clinics 11: 1051-1052.
28. Zhang L (2011) AIDS related knowledge and behavior survey among FSWs in Xinyang City in 2009. Henan J Prev Med 22: 109-110.
29. Zhang H, Chen CG, Lin FH, Xu SY, Yao X, et al. (2011) Analysis of Comprehensive Surveillance Results of HIV/AIDS-related High- risk Groups in Fuzhou City in 2010. Occup and Health 27.
30. Yi JF, Weng DF (2010) Analysis of AIDS sentinel surveillance in Yichun City in 2009. Journal of Qiqihar Medical College 31: 1264-1265.
31. Yan WZ, Zheng KQ, Feng DL, Cai XY, Liu JR, et al. (2010) Analysis of STD/AIDS infection survey among FSWs in Jinghong City in 2009. J Dermatology and Venereology 32: 48-50.
32. Xu XY, Liu SY, Zhang JF (2011) Analysis of AIDS sentinel surveillance in Huhhot in 2009. Journal of Diseases Monitor and Control 5: 393-394+390.
33. Xiang H (2011) Analysis of AIDS related behavior and HIV infection rate survey of FSWs in Laifeng County in 2009. Journal of Mathematical Medicine 24: 199-201.
34. Wu J, He JG (2012) Survey on AIDS-related knowledge and behavior among prostitutes in Jinghu district of Wuhu city, 2011. Anhui J Prev Med 18: 175-178.
35. Wei W (2011) Analysis of the AIDS related behavior and serological survey among FSWs in Xinyang City. Henan J Prev Med 22: 277-278.
36. Wang F, Chu YG, Wang L, Wang FM (2010) Serological Survey And Investigation On AIDS Knowledge And Behaviour Among CSW In Huaiyuan County. Anhui J Prev Med 16: 190-191+202.
37. Wang DL, Zhu YS, Gu ZY, Jiang W, Feng LJ (2012) Analysis of AIDS sentinel surveillance in Zhonglou District in Changzhou City. Jiangsu J Prev Med 23: 47-48.
38. Tang MJ, Zhong FH, Zhang DL, Tan XZ (2012) Analysis of AIDS sentinel surveillance in Yulin City in 2011. Applied Prev Med 18: 40-42.
39. Sun XQ, Tang GX, Mao TS, Zhang YJ, Mao N (2011) 2010 baseline survey among commercial sex workers of Taihe County. CHINA MODERN MEDICINE 18: 151-153.
40. Shao MC, Shen J, Zhao XP, Zhao YQ, Zhang FX, et al. (2010) Investigation on A IDS and Venereal Disease Infection among CSW Crowd in Suzhou Area. Occupation and Health 26: 886-887.
41. Qiu ZH, Dong ZQ, Jin MH, Yang ZR (2012) Sentinel surveillance of AIDS among female sex workers in Huzhou, Zhejiang, 2011. DISEASE SURVEILLANCE 27: 291-293.
42. Miao XL, Cheng HF, Zhang X, Gu J, Ji YY, et al. (2011) Analysis on HIV/AIDS Sentinel Surveillance in Wuxi City in 2010. Occup and Health 27: 2599-2601.
43. Luo MH, Jin WD, Li GB, Wu P (2011) Analysis of surveillance of FSWs in Shaodong County and Shaoyang County in 2009. Practical Preventive Medicine 18: 166-167.
44. Liao MZ, Liu XZ, Kang DM, Fu JH, Wang TZ, et al. (2010) Analysis on the HIV/ AIDS Surveillance Data in Shandong Province in 2009. Pr ev M ed Trib 16: 398-400+403.
45. Li Y, Wu D, Wang SY (2012) Analysis of sentinel surveillance of FSWs in Xiaonan District in 2011. TODAY NURSE.
46. Jing LH, Cai RE, Shi YC, Yu AQ, Wang YB (2010) Results of HIV sentinel surveillance in Yangquan city , 2009. DISEASE SURVEILLANCE 25.
47. JIang DK, Zhang YY, Zhou L, Zhou YJ, ZHong ML, et al. (2010) The Analysis and Investigtion Reports of The Surveillance Program among The Population at High Risk for AIDS. JM ed Pest Control 26.
48. Guo CL, Zhong MR, Yan J, Zhong TQ, Wu YQ (2010) Sentinel surveillance of AIDS among female sex workers in Ruijin city of Jiangxi province, 2009. DISEASE SURVEILLANCE 25.
49. Wen XQ (2010) An analysis Oil Condom Using Behaviors and Influencing factors Among Female Sex Workers(FSW) in Gailin City. Health World 4: 19-20.
50. Nong LP, He B, Li B, Bi SZ (2011) Awareness of HIV/AIDS and infections of HIV, syphilitis and hepatitis C among sex workers of different nationalities in frontier trade area, Guangxi province. Chin J Public Health 27: 1034-1036.
51. Li F, Zhang F, Li RL, Shi L, Zhang ZL, et al. (2006) Study of HIV-risk factors among commercial sex workers in four cities of Xinjiang. Chin J AIDS STD 12.
52. Yang Y, Yao J, Gao M, Su H, Zhang T, et al. (2011) Herpes simplex virus type 2 infection among female sex workers in Shanghai, China. AIDS Care 23 Suppl 1: 37-44.
53. Shi JX, Yan CL (2012) Investigation on intervention results of female sexual workers in Pingshan district in Shenzhen. J Med Pest Control 28: 682-683+686.
54. Hu B, Lin JS, Feng YG, Yang JL, He QY (2004) Analysis of results in popularization the use of condoms in entertainment places for prevention of STD. CHINA TROPICAL MEDICINE 4.
55. He JC, Yi RH, Long QJ, Li N, Yu XF, et al. (2010) Evaluation of the effect of AIDS outreach educational intervention based on sexually transmitted disease clinics for female sex workers. Modern Preventive Medicine 37.
56. He C, CHen L, TAn JG, Shi XD, Gan YX, et al. (2009) Investigation report on comprehensivem onitoring among prostitutes of Shenzhen City in 2008. Chinese Journal of Health Education 25.
57. Xia JH, Guo Y, Dong XY, Zhu XK (2010) Analysis of FSWs surveillance in Tianjin City between 2000 and 2008. Chin J AIDS STD 16: 310+336.
58. Li Y, Detels R, Lin P, Fu X, Deng Z, et al. (2012) Difference in risk behaviors and STD prevalence between street-based and establishment-based FSWs in Guangdong Province, China. AIDS Behav 16: 943-951.
59. Luo J (2005) A survey of AIDS/STD, related knowledge, attitude, behavior and infection rate among FSWs in a city in Guangxi Province. Modern Preventive Medicine 32: 644-645.
60. He JG, Wu ZS, Dou ZD (2005) A Survey on HIV Awareness and Characteristics of Sexua l Behavior among CSWs in Public Places of entertainments in Wuhu City Anhui J PrevMed 11.
61. Zhang M, Rui BL, Xue Q, Qu SH, Wang L (2006) Analysis of AIDS/syphilis surveillance among FSWs in Urumchi City. Chin Prev Med 7: 216-217.
62. Liu YJ, Yu M, Wang BY, Yang Y, Ding HF, et al. (2006) Epidemiological characteristics, sexually transmitted disease and HIV/ AIDS status among 403 female sex workers in Chaoyang district, Beijing. Chin J Drug Depend 15: 401-404.
63. Shi XL (2008) Analyzing Behavior Surveillance of Female Sex Workers in Shehong County from 2006 to 2007. Chinese Health Service Management 25: 492-494.
64. Li WJ, Li Y, Mai RJ, Lin P, Yang LM, et al. (2007) Survey of STD and AIDS knowledge and high risk behavior of female sexual workers in urban area of Yangjiang City. CHINA TROPICAL MEDICINE 7: 1921-1922,1940.
65. Pan GL, Jin Y, Dong XJ (2012) A Survey of Sexually Transmitted Diseases Infection State among 617 Female Sex Workers. Zhejiang Preventive Medicine 24.
66. Cheng XL, Wang FH, Xiao YK (2009) Analysis on the Data of HIV Sentinel Surveillance in 2002-2006 in Anhui Province. Anhui J PrevMed 15.
67. Liu C, Liu J, Zhou W, Yang LD, Yao ZZ, et al. (2011) Baseline investigation on three kinds of high risk population in Wuhan, China-Bill& MelindaGates Foundation AIDS program. J of Pub Health and Prev Med 22: 15-18.
68. Weng YQ, Bai Y, Feng WD (2011) High risk behavior toward HIV/AIDS of female sexual service workers with different marital status inLiuzhou City. CHINA TROPICAL MEDICINE 11: 170-171.
69. Lu F, Jia Y, Sun X, Wang L, Liu W, et al. (2009) Prevalence of HIV infection and predictors for syphilis infection among female sex workers in southern China. Southeast Asian J Trop Med Public Health 40: 263-272.
70. Zhang YH, Bao YG, Li CM, Han L, Sun JP, et al. (2011) Study of HIV/ syphilis infection status of commercial sex workers in 15 cities of China. Chin Prev Med 12: 387-390.
71. Shi WY, Xie YY, Liu C (2012) Behavioral and serological surveillance among female sex workers in Fengtai district, Beijing from 2006 to 2009. Chin J Public Health 28: 109-110.
72. Dong XY, Zhu XK (2008) Surveillance report of AIDS high-risk population in Tianjin in 2006. DISEASE SURVEILLANCE 23: 31-33.
73. Zi GS, Yao HB, Cha XS, Guo JF, Zi ZH (2009) Investigation on the Status Related to AIDS among Female Sex Workers in Weishan County in 2008. Prev Med Trib 15: 1092-1093.
74. Zhou JH, Huang ZM, Deng BQ, Chen Y, Luo RH (2010) SURVEY ON THE BEHAVIORAL CHARACTERISTICS AND SEXUALLY TRANSMITTED INFECTION AMONG 418 FEMALE COMMERCIAL SEX WORKERS. Modern Preventive Medicine 37: 1158-1159,1161.
75. Zhou CX, Pan ZP, Chen ZY, Guo HJ (2011) Results analysis of AIDS sentinel surveillance in Zunyi city, 2010. Jiangsu J Prev Med 22: 11-13.
76. Zheng WA, Zhong N, Zhu HH, Wang FQ, Lu YZ, et al. (2009) Analysis of the Monitoring Results of 1144 Unlicensed Prostitutes for Infection of Hepatitis B Virus( HBV) , Human Immunodeficiency Virus( HIV) and Syphilis. The Chinese Journal of Dermatovenereology 23: 502-503.
77. Zhao SH, Wang JJ, Fang ZH, Yan TQ, He W, et al. (2007) AIDS/STD serological and behavioral survey among 140 FSWs. Practical Preventive Medicine 14: 1926-1927.
78. Zhao JZ, Ren SH, Wan Y, Xu H, Zhou T, et al. (2011) Research on AIDS/Infectious Diseases of Genital Tract and Related Behavior of Female Sex Workers in Low-grade Areas. Chinese Journal of Social Medicine 28: 326-328.
79. Zhang YX, Lin HT, Feng WD, Shan GS, Zhang TJ (2011) Syphilis and HIV infection status among commercial sexual workers in Liuzhou, Guangxi. J Trop Med 11: 337-339+355.
80. Zhang Y, Zhou J, Xie JY, Zhang QS, Wang Q, et al. (2005) Investigation on knowledge, awareness and risk behaviour related to HIV/ AIDS among female sex workers based on lowclass establishments in some areas of China. Chin J AIDS STD 11: 415-417.
81. Zhang QQ, Zhang XP, Yang HT, Xiao ZP, Zhou JB, et al. (2012) Prevalence of sexually transmitted disease and risk factors among female sex workers in Jiangsu province. ACTA UNIVERSITATIS MEDICINALIS NANJING (Natural Science) 32: 473-478.
82. Zhang CP (2008) Surveillance report of HIV and syphilis among the commercial sex workers in the entertainment establishments in Gucheng District. Soft Science of Health 22: 269-270.
83. Yu KW, Wang CQ, Qiu GP, Li XC, Wang LY, et al. (2010) Results from Sentinel Surveillance of H IV /AIDS Among Commercial Sex Workers in Lancang County in 2009. Practical Preventive Medicine 17: 2303-2305.
84. Yang JY, Shen XW (2011) Status of STDs Epedemic Among Female Sexual Workers in Chong’an Distric in Wuxi. J Prev Med Inf 27: 905-907.
85. Yang BF, Xu J, Yao ZZ, Chen ZD, Wang X, et al. (2006) An analysis of the behavior characteristics and the infection of HIVand syphilis among the commercial female sex workers. Chin J Dis Control Prev 10: 406-408.
86. Xu YF, Zhou FH, Mo XJ, Li SS, He Y, et al. (2009) Surveillance of Commercial SexWorkers in Nanning, 2007- 2008. J Prev Med Inf 25: 615-617.
87. Xu SM, Qian TX, Peng CL, Yang MF, Lu RR (2006) AIDS/syphilis surveillance in Chongqing City in 2004. Modern Preventive Medicine 33: 833-834.
88. Xu HF, Song T, Xie YK, Gao LD, Lv JY, et al. (2011) Effectiveness and analysis of interventions for HIV/AIDS prevention among female sex workers in entertainment establishments in Shangqiu. Henan J Prev Med 22: 95-96.
89. Xu JG (2009) A survey of 407 HIV/syphilis infected cases of specific groups in Yandu district of Yancheng City. China Prac Med 4: 239-241.
90. Xia DY, Li GY, Lu HY, Zhao YJ, Wang J (2009) Prevalence rate of HIV infection and syphilis as well as factors associated with syphilis among female sex workers in Beijing, 2006-2008. Chin J AIDS STD 15: 601-603+606.
91. Wu CL, Zhang JH, Fan SF, Wei DY, Li CX, et al. (2010) A STUDY ON HIV/SYPHILIS PREVALENCE AND RELATED KNOWLEDGE AND BEHAVIORS AND THEIR INFLUENCE FACTORS AMONG FEMALE COMMERCIAL SEX WORKERS IN A CITY. Modern Preventive Medicine 37: 499-501.
92. Wei ZY, Sun L (2010) Investigation on Behavior and Infection of STDs and AIDS among Female Sex Workers at Entertainment Sites in Yangzhou City, 2009. Prev Med Trib 16: 1123-1125.
93. Wei QH, Shan GS, Feng WD (2008) Analysis of Serobgical and Behavioral Survey Results for HIV and TP in Female Sex Workers(FSW) in 2005. Contemporary Medicine: 61-62.
94. Wang WM, Xue LJ, Xia LM, Tang QF, Shen L (2008) Survey of 297 sex workers and the infectious status of STD and HIV/AIDS. CHINA TROPICAL MEDICINE 8: 827-828.
95. Wang WW, Yu SL, Lin GL (2010) Analysis on the data of HIV high-risk groups sentinel surveillance in 2009 in Neijiang City. J Prev Med Inf 26: 309-311.
96. Wang JH (2010) A survey of HIV, HCV, HBV and syphilis infection rate among high-risk groups in a county in Shandong Province. Chinese Journal of Social Medicine 8: 22-23.
97. Wang JY, Wang T, Cen YZ, Lai XH, Li L, et al. (2010) Prevalence of Sex Transmitted Disease or Its Related Symptoms and Associated Risk Factors among Female Sex Workers in Zhongshan. Journal of Tropical Medicine 10: 477-480.
98. Wang GX, Ding GW, Wang HB, Xu JJ, Chang DF, et al. (2008) Two cross-sectional survey of HIV/STD infection of FSWs in a city of Yunnan. Soft Science of Health 22: 343-345.
99. Wang BF, Chen QJ, Zhang YH, Chen YL, Li SR (2004) Epidemiological analysis of behavior characteristics and HIV/syphilis infection rate among FSWs DISEASE SURVEILLANCE: 296-297.
100. Wang B, Wang J (2010) AIDS/syphilis Surveillance among 400 female commercial sex workers. J Dermatology and Venereology 32: 47-48.
101. Wang HB, Wang N, Ma JG, Wang GX, Chang DF, et al. (2007) Study On the association between Vaginal douching and sexually transmitted diseases among Female Sex Workers in a county of Yunnan province. Chin J Epidemiol 28: 558-561.
102. Wang FH, Cheng XL, Su B, Ji GP (2009) ANALYSIS OF THE RESULTS OF THE COMPREHENSIVE H IV /AIDS SURVEILLANCE AMONG SEX WORKER S IN ANHUI IN 2008. Anhui J Prev Med 15: 407-408.
103. Tao LD, Qi YJ, Wei HW, Chen JJ (2011) Epidemiology of HIV and Syphilis Among Female Sex Workers in Chenguan County, Gansu Province. CHINESE PRIMARY HEALTH CARE 25: 65-66.
104. Tang YQ, Ma SB, Zhang J (2007) Analysis of HIV/syphilis infection rate and related behavior survey among FSWs in Changping District in Beijing City. Chin J AIDS STD 13: 575-576.
105. Tang MJ, Zhang DL, Zhong FH, Liu J, Chen J, et al. (2009) Behavior survey of 362 FSWs in Yunlin City Practical Preventive Medicine 16: 118-119.
106. Tan Y, Feng L (2011) STDs/AIDS Sentinel Surveillance of FSW in Ganzi Prefecture, 2006 and 2010. J Prev Med Inf 27: 670-673.
107. Tan SN, Lan RW, Wei JZ, Xie SY, Tan Q, et al. (2010) STD/AIDS infection survey among FSWs in Laibin City in 2007. Applied Prev Med 16: 98-99.
108. Tan JG, Chen L, Cai WD, Yang ZR, Shi XD, et al. (2009) STUDY ON AIDS / STDS RELATED RISK FACTORS AND BEHAVIORS AMONG PROSTITUTE IN SHENZHEN. Modern Preventive Medicine 36: 3146-3147+3153.
109. Tan WW, Zhou FH, Liu HY, Mo XJ, Luan Y (2008) Analysis of comprehensive surveillance results of FSWs in Nanning City in 2007. Guangxi Medical Journal 30: 1727-1728.
110. Suo JM, Zhang XQ, Tian Q, Li JL, Guo QH (2011) HIV/syphilis infection and AIDS related knowledge and behavior survey among FSWs in Changzi County in 2010. Jiankang Bidu 8: 142-143.
111. Sun ZX, Xie FH, Lin SF, Wen MQ (2008) Survey of knowledge and behavior associated with AIDS in prostitutes in educational house. CHINA TROPICAL MEDICINE 8: 511-512.
112. Song SJ, Chen SC, Ding JM, Cheng J, Luo Y, et al. (2008) ANALYSIS OF THE STATUS OF STD/AIDS DETECTION AND CONDOM UTILIZATION OF 272 CSW IN HANGZHOU. Modern Preventive Medicine 35: 3625-3626.
113. Shi WY, Bai JM, Li JJ, Qu YM (2007) A survey on KAB of AIDS among 114 female commercial sex workers in Fengtai District in Beijing. Chinese Journal of Health Education 23: 120-122.
114. Ren XY, Bo FB, Bao ZQ, Xu RQ, Zhou BP, et al. (2006) AIDS/syphilis infection rate and related behavior survey among FSWs in Hohhot City. Chin J AIDS STD 12: 551-552.
115. Qiu JJ, Wang ZZ, Li LL, Liu Y (2008) Survey on AIDS Related Know ledge and Behavior Among Prostitutes in Shenzhen. J Prev Med Inf 24: 876-878.
116. Qin YM, Wang XH, Shi XD, Tan JG, Liu G, et al. (2010) Analysis of HIV and Syphilis Infection among Sex Workers, Drug Abusers and Container Drivers in Shenzhen. Journal of Tropical Medicine 10: 889-891.
117. Qi HZ (2007) Study of STD/AIDS detection and condom utilization conditions of 207 unlicensed prostitutes in Yancheng City. Disease Surveillance 22: 31-32.
118. Pan XL, Liu ZH, Chen FX, Liang X (2008) AIDS/syphilis related high-risk behavior survey among FSWs in Baise City between 2005 and 2006. Journal of Applied Preventive Medicine 14: 287-289.
119. Pan XL (2009) The Investigation of Venereal Disease for 864 Female Sex Workers. Youjiang Medical Journal 37: 10-13.
120. Ouyang N, Xu FJ (2010) Analysis of AIDS Comprehensive Surveillance Results among FSWs in Dongchuan District of Kunming City in 2009. Soft Science of Health 24: 471-473.
121. Ni MJ, Liu YH, Cheng J, Wang DL, Dong YH, et al. (2005) A comprehensive survey on HIV/ AIDS in Kashgar prefecture of Xinjiang. Chin J AIDS STD 11: 353-356.
122. Mei L, Bai SM, Liu JH, Gao LJ, Wang HJ, et al. (2009) A survey of STD infection rate and intervention effectiveness among FSWs in four cities in Shanxi Province. Chin J AIDS STD 15: 528-529.
123. Luo Y, Chen SC, Ding JM, Cheng J, Xu K, et al. (2008) Analysis of HIV/AIDS sentinel surveillance in Hangzhou. Disease Surveillance 23: 717-719.
124. Luo J (2005) Analysis of the Results from HIV Surveillance of Unlicensed Prostitutes. Disease Surveillance 20: 409-412.
125. Lu H, Zhang LJ, Li SL (2003) AIDS/STD related knowledge and behavior survey among druggers and FSWs in Urumchi City. Endemic Diseases Bulletin 18: 52-53.
126. Liu XZ, Liao MZ, Fu JH, Su SL, Huang T (2006) Analysis on HIV/ AIDS Surveillance Data of Shandong Province in 2004. Prev Med Trib Vol 12: 80-82.
127. Liu WB (2010) Analysis of the AIDS/STD serological and behavior survey among FSWs in Tonghua City Contemporary Medicine 16: 155+135.
128. Liu LR, Liu M, Lu HY, Xia DY (2007) Analysis of HIV/AIDS related risk behaviors among female sex workers at entertainment establishments in two districts of Beijing. Chin J AIDS STD 13: 532-535.
129. Liu CQ, Hou WJ, Kong XS, Su J, Xi JM, et al. (2010) Analysis of intervention effectiveness of collaborative project on AIDS high-risk groups in Chengjiang County. Soft Science of Health 24: 335-338.
130. Liao MZ, Liu XZ, Fu JH, Qian YS, Wang XY (2008) Analysis of HIV/ AIDS Surveillance Data in Shandong Province in 2007. Pr ev M ed Trib 14: 1143-1145.
131. Liang SP, Zhang SY (2010) Evaluation of AIDS/STD intervention effectiveness among FSWs in Huinong District JM ed Pest Control 26: 1155-1156.
132. Li XY, Zhang YL, Zhang SP, Zhu ML, Ma L, et al. (2011) Infection of sexually transmitted diseases among 98 female sex workers. Chin J Nosocomiol 21: 2274-2275.
133. Li YF, Shen JZ, Yao WW (2011) Intervention Effectiveness of AIDS Awareness and Behavior among Female Sex Works in Qingpu District of Shanghai City. Occup and Health 27.
134. Li Y, Lin P, Roger D, Fu XB, Deng ZM, et al. (2009) Prevalence of HIV infection and sexually transmitted diseases and associated risk factors among female sex workers in Guangdong province. DISEASE SURVEILLANCE 24.
135. Li XJ, Zhang XP, Hu ZW, Fang D, Li ZR (2006) Analysis of the Results from Sentinel Surveillance of HIV infection in Hefei City. Anhui J Prev Med 112.
136. Li WZ (2009) HIV/AIDS surveillance report among commercial sexual workers in Jianshui County. Soft Science of Health 23.
137. Li JX, Ruan SM, Yang H, Zhu YW, Zhang CW, et al. (2007) An Analysis of HIV Infection and Risk Behavior of Commercial Sex Workers in Ji'nan City from 2003 to 2005. Prev Med Trib 13.
138. Li F, Ma SB, Liu HX (2012) Analysis of AIDS sentinel surveillance of Changping District in Beijing, 2011. Chinese Journal of Health Education 28.
139. Lei ZQ, Du MR, Wang Z, Zhong XD (2005) Analysis of Serological and Behavior al Survey Results for HIV and TP in 259 Female Sex Worker s (FSW) from Communities in 2004. DISEASE SURVEILLANCE 20.
140. Jin YL, Yao YS, Ye DQ, He JG, Dou ZD, et al. (2009) Investigation and analysis on condom use status among female commercial sex workers. Chinese Journal of Disease Control & Prevention 13.
141. Ji CH, Ren XP, Shen JP, Zhao LH, Feng Y, et al. (2009) Analysis of STD and AIDS of Commercial Sex Workers in Entertainment Places in Gaoping City from 2004 to 2006. Prev Med Trib 15.
142. Huang KZ, Chen XH, Li WJ, Chen ZF (2010) A AIDS/STD knowledge and high-risk behavior survey among FSWs in Yangjiang City. South China J Prev Med 36: 41-42.
143. Huang JF, Li CS, Hu SX, Huang JY, Xuan RL, et al. (2010) HIV/AIDS-related knowledge and behavior in female sex workers in Qingyuan City. South China J Prev Med 36: 18-21.
144. Huang GL, Zhai QX (2012) AIDS related knowledge and risk behaviors investigation among the female commercial sex works in a county of Guangxi. J Med Pest Control 28.
145. Hu XQ, Yan XY, Yang PR, Tian H, Li Z, et al. (2011) Analysis of surveillance of AIDS high-risk groups in a city in Shannxi Province in 2010. J Prev Med Chin PLA 29.
146. Gao LL, Che ZM, Lu Y (2008) A cross- sectional study on STDs/AIDS and HIV of 270 female sex wokers. J Derm a tology and Venereology 30.
147. Gao JM (2008) Analysis on results of STD and AIDS surveillance during 2006 in Huairou of Beijing. Occup and Health 24.
148. Feng N (2011) Analysis on sentinel surveillance of female sex workers in Datong City in 2010. Medical Information 24.
149. Du JQ, Wang GX, Wang WZ, Gu J, Chang DF, et al. (2008) Analysis on the survey of HIV/AIDS/STI among the commercial sex workers in Kaiyuan City. Soft Science of Health 22.
150. Dong XY, Zhou N, Guo Y, Yu MH (2009) Analysis on sentinel surveillance of female sexual workers and their clients in Tianjin City ,2007. South China J PrevMed 35.
151. Ding XB, Yi HR, Jiang XF, Han LX, Wu GH, et al. (2006) Analysis of status of AIDS related knowledge ,attitude and risk behavior among 519 female sex workers in Chongqing. Chin J AIDS STD 12.
152. Dai XN, Tang SK, Huang XM, Cao WL, Deng HY, et al. (2009) Investigation and Analysis on Sexually Transmitted Infection Among Different Females Group in Guangzhou. Journal of Tropical Medicine 9.
153. Cui YZ, Zhu L, Yuan LL, Zhao YS, Wang J (2011) AIDS knowledge, behavior and HIV/ syphilis infection survey among FSW in Harbin from 2006 to 2009. Chin J AIDS STD 17.
154. Chen YL, Cai XH, You TZ, Chen QJ (2009) Analysis on Risk Behaviors and HIV/ Syphilis Infection among Female Sex Workers( FSWS) in Detention Home of Longyan City from 2001 to 2007. Prev Med Trib 15.
155. Chen W, Zhou Y, Zhou MR, Zhang ZK (2011) AIDS infection and high-risk behavior survey among FSWs in Guilin City in 2009 Applied Prev Med 17.
156. Chen SX, Zhang ML, Han XM (2011) Survey on AIDS Related Knowledge, Behavior and the HIV Infection Status Among Commercial Sex Workers in Gaomi City, 2009. Prev Med Trib 17.
157. Chen SP, Tu BY, Wang X (2010) Survey and Analysis on HIV/AIDS-Related Behavior and Recognition among HIV/AIDS High-risk Population of Xunyang District Jiujiang City. Chin J Evid-based Med 10.
158. Chen MF, Xu FF, Qiu LX (2011) Chinese Journal of Health Laboratory Technology. Chinese Journal of Health Laboratory Technology 21: 1266-1267.
159. Chen L, Tan JG, Shi XD, Gan YX, Zhang Y, et al. (2010) The Comprehensive Surveillance of AIDS among Unlicensed Female Sex Workers in Shenzhen City. Journal of Tropical Medicine 10.
160. Chen L, Tan JG, Luo XR, Shi XD, Gan YX, et al. (2009) A Report on the Monitoring of AIDS among the Sex Workers in Shenzhen. Journal of Tropical Medicine 9.
161. Chen L, Feng TJ, Tan JG, Shi XD, Wang XH, et al. (2006) Analysis of HIV/AIDS sentinel surveillance in Shenzhen. Chin J AIDS STD 12: 136-139.
162. Chen FC, Zhao Y, Xiao HT, Tong XY (2011) Analysis of HIV/AIDS sentinel surveillance of FSWs in Dangyang City in 2010. J of Pub Health and Prev Med 22.
163. Cao XY, Jiang ZQ, Ruan YH, Liang S, Qin GM, et al. (2006) Sexually transmitted diseases and risk factors among female sex workers in a heavy drug using area. Chin J AIDS STD 12.
164. Cao JX, Kang YF, Jin LH (2008) Analysis on survillance results of AIDS shots and other groups with high risk behaviors in Yangquan City in 2003-2006. Chinese Journal of Health Education 24.
165. Cao H (2010) Analysis of high risk AIDS-related behavior characteristics among 186 female sex workers at Xiqing district of Tianjin. Port Health Control 15.
166. Cai Y, Zhao JX, Lu JB, Gao LM, Chen LY, et al. (2010) Data Analysis of Sentinel Surveillance of AIDS/ STD among Female Sex Workers ( FSW) in Yuxi City in 2005 and 2009. Prev Med Trib.
167. Bo FB, Gao P, Yun ZP, Ren XY, Liu XP, et al. (2007) An AIDS epidemiological survey of female sex workers crowd in Hohhot. Inner Mongolia Med J 39: 971-973.
168. Bai Y, Zhang YX, Cui XL (2010) Survey of knowledge behaviors and HIV infection among commercial sex workers from high, middle and low level places. Chin J Dis Control Prev 14.
169. Bai JM, Shi WY, Zhu LY, Qu YM (2006) Analysis of behaviors and related-diseases sureillance of 114 female sexual workers in fengtai in Beijing. Chin J AIDS STD 12.
170. Zhu BY, Bu J, Huang PY, Zhou ZG, Yin YP, et al. (2012) Epidemiology of sexually transmitted infections, HIV, and related high-risk behaviors among female sex workers in Guangxi Autonomous Region, China. Jpn J Infect Dis 65: 75-78.
171. Xu JJ, Wang N, Lu L, Pu Y, Zhang GL, et al. (2008) HIV and STIs in clients and female sex workers in mining regions of Gejiu City, China. Sex Transm Dis 35: 558-565.
172. Ruan YH, Cao X, Qian HZ, Zhang L, Qin G, et al. (2006) Syphilis among female sex workers in southwestern China: potential for HIV transmission. Sex Transm Dis 33: 719-723.
173. Liao MZ, Nie X, Pan R, Wang C, Ruan S, et al. (2012) Consistently low prevalence of syphilis among female sex workers in Jinan, China: findings from two consecutive respondent driven sampling surveys. PLoS One 7: e34085.
174. Chen XS, Yin YP, Liang GJ, Gong XD, Li HS, et al. (2005) Sexually transmitted infections among female sex workers in Yunnan, China. AIDS Patient Care STDS 19: 853-860.
175. Zhao YQ, Zhao XP, Cao XP, ZhaNG FX, Shao MC (2010) High-risk behavior and infection rate survey of AIDS/STD among 396 FSWs. Shanghai Journal of Preventive Medicine 22: 137+141.
176. Wei XW, Wang H, Su P, Wang CC, Feng XR (2002) STD/ HIV surveillance of the waitresses in the public of entertainment. Chinese Journal of Disease control and Prevention 6: 303-305.
177. Qi HZ (2003) Analysis on The Investigation Results of STD among Prostitutes. Disease Surveillance 18: 45-47.
178. Ye YQ, Wei XW, Wang H, Su P (2005) AIDS/STD Surveillance among FSWs in Shihezi in 2003. Endemic Diseases Bulletin 20: 39-40.
179. Xue FH (2006) A survey on HIV infection and risk behavior information among sex workers in entertainment sites. Chin J Dis Control Prev 10: 526-528.
180. Luo Z, Zha YF, Huang ZM (2007) Analysis of FSWs surveillance in Songjiang District in Shanghai City in 2006. Shanghai Journal of Preventive Medicine 19: 184-185.
181. Ao X, Han QY (2008) Survey of AIDS-related knowledge, behaviors and infection with HIV and syphilis among 105 sex workers. DISEASE SURVEILLANCE 23: 714-716.
182. Huang YK, Li MQ, Luo LR, Wu YX (2008) Study on the effect of interventions for preventing HIV among commercial female sex workers in entertainment establishments. Modern Preventive Medicine 35: 2328-2329.
183. Luo XY, Shi RQ (2010) A survey of HIV/syphilis infection rate among FSWs in Pinghu City. Zhejiang Preventive Medicine 22: 25-26.
184. Zhang GS, Ou HH, Lan L (2009) Analysis on AIDS knowledge, behavior and HIV infection among 512 female sexual workers. South China J Prev Med 35: 22-24+27.
185. Yin FL, Shi GZ, Zhang Y, Xiang MY (2009) AIDS related knowledge, behavior and serological survey among FSWs in Jiading District in Shanghai City. Shanghai Journal of Preventive Medicine 21: 484-485.
186. Luo L, Zhou S, Wang Y (2009) STUDY AND ANALYSIS ON THE AIDS RELATED BEHAVIOR OF FEMALE SEXUAL WORKERS IN THE LOW -GRADE ENTERTAINMENT PLACES. Modern Preventive Medicine 36: 3972-3973+3975.
187. Chen JQ, Chen WY (2009) Analysis of STD infection among 168 FSWs Zhe jiang Prev Med 21: 25-29.
188. Xia GM, Zhang YJ, Sun XQ (2008) Analysis of Comprehensive Surveillance Results of FSWs in Taihe County in 2006. Anhui J Prev Med 14: 70-71.
189. Luo XR (2008) Knowledge, Behavior About STD /AIDS and HIV Infection Situation of Female Commercial Sex Workers in Yibin City. J Prev Med Inf 24: 566-568.
190. Chen YH (2012) Analysis of syphilis and HIV test results in 400 female sex workers in Rugao city. Chinese Journal of Health Laboratory Technology 22.
