# Supplementary material for: A Decline in HIV and Syphilis Epidemics in Chinese Female Sex Workers (2000–2011): A Systematic Review and Meta-Analysis
Source: PLoS One. 2013 Dec 13;8(12):e82451. doi: 10.1371/journal.pone.0082451 (PMC3862622; doi:10.1371/journal.pone.0082451)
Supplement: Table S4 — Data extraction table of Chinese female sex workers in different workplaces. (DOC) [file pone.0082451.s004.doc]

**Table S4. Data extraction table of Chinese FSWs in different workplaces.**

| **First author**^†^ | **Year of publication** | **Study period** | **Province** | **Workplace**^*^ | **HIV testing cases** | **Prevalence of HIV %** | **Syphilis testing cases** | **Prevalence of syphilis %** |
| --- | --- | --- | --- | --- | --- | --- | --- | --- |
| Li Dongmin^1^ | 2007 | 2006 | Guizhou | Hotels and Karaoke Halls | 220 | 0.00 | 220 | 3.20 |
|  |  |  |  | Salons | 110 | 0.91 | 110 | 4.50 |
|  |  |  |  | Street-based | 102 | 0.00 | 102 | 43.10 |
| Peng Hua^2^ | 2008 | 2007 | Guangdong | Rental houses and Street-based | 190 | 0.52 | 190 | 31.60 |
| Yang Ping^3^ | 2009 | 2007 | Guangdong | Salons, Leisure centers and Massage parlors | 190 | 0.00 | 190 | 7.90 |
|  |  |  | Guangdong | Rental houses and Inns | 97 | 1.03 | 97 | 21.60 |
|  |  |  | Guangdong | Lanes, Lakefront and Street-based | 127 | 0.00 | 127 | 37.80 |
|  |  |  | Hainan | Salons, Leisure centers and Massage parlors | 182 | 0.00 | 182 | 9.90 |
|  |  |  | Hainan | Rental houses and Inns | 44 | 4.55 | 44 | 20.50 |
|  |  |  | Hainan | Lanes, Lakefront and Street-based | 102 | 0.00 | 102 | 38.20 |
| Miao Xiangfen^4^ | 2009 | 2008 | Hebei | Rural Entertainment venues | 250 | 0.00 | 250 | 3.20 |
| Jiang Nan^5^ | 2012 | 2010 | Henan | Street-based | 475 | 0.21 | 475 | 0.84 |
| Xiang Hong^6^ | 2011 | 2009 | Hubei | Night clubs, Hotels and Karaoke halls | 146 | 0.00 | 146 | 0.68 |
|  |  |  |  | Dancing halls, Bars, Massage parlors and Bath centers | 135 | 0.00 | 135 | 2.96 |
|  |  |  |  | Street-based | 129 | 0.00 | 129 | 6.98 |
| Yan Li^7^ | 2012 | 2007 | Guangdong | Bars, Night clubs, Massage parlors, Karaoke halls and Hotels | 241 | 0.00 | 241 | 5.70 |
|  |  |  |  | Street-based | 77 | 0.00 | 77 | 15.60 |
| Pan Ganglei^8^ | 2012 | 2011 | Zhejiang | Hotels and Dancing halls | 88 | 0.00 | 88 | 5.68 |
|  |  |  |  | Salons | 255 | 0.00 | 255 | 13.33 |
|  |  |  |  | Street-based | 274 | 0.92 | 274 | 15.69 |
| Zhao Jinzhu^9^ | 2011 | 2009 | Hubei | Salons, Footbath rooms and Leisure centers | 163 | 0.00 | 165 | 8.50 |
| Zhang Yingxia^10^ | 2011 | 2007 | Guangxi | Night clubs, Hotels, Karaoke halls, Dancing halls, Bars, Massage parlors and Bath centers | 317 | 0.00 | 317 | 1.60 |
|  |  |  |  | Street-based | 86 | 0.00 | 86 | 5.80 |
| Zhang Yan^11^ | 2005 | 2004 | Shandong | Street-based | 180 | 0.00 | 180 | 0.28 |
| Wang Jinghua^12^ | 2010 | 2009 | Shandong | Hotels, Karaoke halls, Bath centers | 298 | 1.01 | 298 | 1.01 |
|  |  |  |  | Street-based | 50 | 0.00 | 50 | 10.00 |
| Tan Jingguang^13^ | 2009 | 2008 | Guangdong | Hotels and Night clubs | 99 | 0.00 | 99 | 5.10 |
|  |  |  |  | Massage parlors, Karaoke halls and Bars | 88 | 0.00 | 88 | 0.00 |
|  |  |  |  | Street-based | 148 | 0.00 | 148 | 2.00 |
| Pan Xinlian^14^ | 2008 | 2006 | Guangxi | Hotels and Karaoke halls | 104 | 0.00 | 104 | 0.96 |
|  |  |  |  | Salons | 114 | 0.00 | 114 | 3.51 |
|  |  |  |  | Rental houses | 35 | 5.71 | 35 | 14.29 |
| Bai Yu^15^ | 2010 | 2008 | Guangxi | Hotels, Night clubs and Karaoke halls | 431 | 0.23 | 431 | 1.39 |
|  |  |  |  | Salons and Footbath rooms | 449 | 0.45 | 449 | 1.34 |
|  |  |  |  | Rental houses and Street-based | 167 | 0.60 | 167 | 9.58 |
| Luo Xiaoying^16^ | 2010 | 2009 | Zhejiang | Karaoke halls and Bath centers | 350 | 0.29 | 350 | 0.86 |
|  |  |  |  | Street-based | 180 | 0.00 | 180 | 5.56 |
| Zhong Jian^17^ | 2011 | 2009 | Guangxi | Hotels and Leisure centers | 45 | 0.00 | Unavailable | Unavailable |
|  |  |  |  | Salons, Massage parlors, Leisure centers | 648 | 0.62 | Unavailable | Unavailable |
|  |  |  |  | Street-based | 473 | 1.27 | Unavailable | Unavailable |

Seventeen articles were included with 40 records of HIV prevalence (medium and high-tier 21, low-tier 19) and 37 records of syphilis prevalence (medium and high-tier 19, low-tier 18).

^*^Categorized into two groups: Medium and high-tier (Hotels, Karaoke Halls, Salons, Leisure centers, Massage parlors, Night clubs, Dancing halls, Bars, Bath centers and Footbath rooms) and Low-tier (Street-based, Rental houses, Inns, Lanes, Lakefront and Rural entertainment venues).

^†^References:

1. Li DM, Yuan F, Hu SY, Lu F (2007) High risk behaviors and HIV/STI prevalence among female sex workers in different settings. Chin J AIDS STD 13.
2. Peng H, Yang LG, Zhang MM, Wang H, Huang XX, et al. (2008) AIDS/STD knowledge, attitude and behavior survey among 60 street-based FSWs. Chin J AIDS STD 14: 628-629.
3. Yang P, Wang QQ, Peng H, He L (2009) A survey of syphilis and HIV infection in medium-low-income female sex workers. China J Lepr Skin Dis 25: 174-176.
4. Miao XF, Zhao HR, Li QM, Li JJ, Zong XM, et al. (2009) The Character of Commercial Sex Workers and HIV/Syphilis/HBsAg Infection in Low - grade P lace of Rural Areas. Chin J Pest Control 25: 813-815.
5. Jiang N (2012) Surveillance of risk behawiors facilitating among commercial sex works and analysis of HIV, Syphilis, HCV and HBV infection. J Medical Forum 33.
6. Xiang H (2011) Analysis of AIDS related behavior and HIV infection rate survey of FSWs in Laifeng County in 2009. Journal of Mathematical Medicine 24: 199-201.
7. Li Y, Detels R, Lin P, Fu X, Deng Z, et al. (2012) Difference in risk behaviors and STD prevalence between street-based and establishment-based FSWs in Guangdong Province, China. AIDS Behav 16: 943-951.
8. Pan GL, Jin Y, Dong XJ (2012) A Survey of Sexually Transmitted Diseases Infection State among 617 Female Sex Workers. Zhejiang Preventive Medicine 24.
9. Zhao JZ, Ren SH, Wan Y, Xu H, Zhou T, et al. (2011) Research on AIDS/Infectious Diseases of Genital Tract and Related Behavior of Female Sex Workers in Low-grade Areas. Chinese Journal of Social Medicine 28: 326-328.
10. Zhang YX, Lin HT, Feng WD, Shan GS, Zhang TJ (2011) Syphilis and HIV infection status among commercial sexual workers in Liuzhou, Guangxi. J Trop Med 11: 337-339+355.
11. Zhang Y, Zhou J, Xie JY, Zhang QS, Wang Q, et al. (2005) Investigation on knowledge, awareness and risk behaviour related to HIV/ AIDS among female sex workers based on lowclass establishments in some areas of China. Chin J AIDS STD 11: 415-417.
12. Wang JH (2010) A survey of HIV, HCV, HBV and syphilis infection rate among high-risk groups in a county in Shandong Province. Chinese Journal of Social Medicine 8: 22-23.
13. Tan JG, Chen L, Cai WD, Yang ZR, Shi XD, et al. (2009) STUDY ON AIDS/STDS RELATED RISK FACTORS AND BEHAVIORS AMONG PROSTITUTE IN SHENZHEN. Modern Preventive Medicine 36: 3146-3147+3153.
14. Pan XL, Liu ZH, Chen FX, Liang X (2008) AIDS/syphilis related high-risk behavior survey among FSWs in Baise City between 2005 and 2006. Journal of Applied Preventive Medicine 14: 287-289.
15. Bai Y, Zhang YX, Cui XL (2010) Survey of knowledge behaviors and HIV infection among commercial sex workers from high, middle and low level places. Chin J Dis Control Prev 14.
16. Luo XY, Shi RQ (2010) A survey of HIV/syphilis infection rate among FSWs in Pinghu City. Zhejiang Preventive Medicine 22: 25-26.
17. Zhong J, Lin J, Hu YM, Tan LL, Wang G (2011) HIV/STD infection and risk behaviors among commercial sex workers at various places in Wuzhou City, Guangxi Zhuang Autonomous Region. Chinese Journal of Health Education 27: 177-180.
