# Supplementary material for: A Decline in HIV and Syphilis Epidemics in Chinese Female Sex Workers (2000–2011): A Systematic Review and Meta-Analysis
Source: PLoS One. 2013 Dec 13;8(12):e82451. doi: 10.1371/journal.pone.0082451 (PMC3862622; doi:10.1371/journal.pone.0082451)
Supplement: Table S5 — Results of subgroup analysis and meta-regression. (DOC) [file pone.0082451.s005.doc]

**Table S5. Results of subgroup analysis and meta-regression.**

|  | **HIV** | | | **Syphilis** | | |
| --- | --- | --- | --- | --- | --- | --- |
| **Subgroup (No. of datasets)** | **Prevalence % (95% CI)** | ***I*^2^ (%)** | ***p*-value from meta-regression**|| | **Prevalence % (95% CI)** | ***I*^2^ (%)** | ***p*-value from meta-regression**|| |
| **Language** | | | | | | |
| Chinese (328) | 0.171 (0.145, 0.198) | 47.300 | Reference | 4.319 (4.061, 4.577) | 93.900 | Reference |
| English (13) | 2.43 (1.588, 3.302) | 96.400 | **0.002** | 7.922 (5.814, 10.031) | 91.800 | 0.796 |
| **Sample size** | | | | | | |
| < 200 (63) | 0.401 (0.267, 0.535) | 0.000 | Reference | 6.626 (5.534, 7.718) | 91.700 | Reference |
| 200-400 (169) | 0.201 (0.164, 0.238) | 0.000 | 0.230 | 4.809 (4.399, 5.219) | 94.000 | 0.997 |
| ≥ 400 (109) | 0.171 (0.129, 0.213) | 84.000 | **0.016** | 3.810 (3.446, 4.174) | 95.200 | 0.541 |
| **Study location** | | | | | | |
| Entertainment Venues (259) | 0.192 (0.160, 0.225) | 67.700 | Reference | 3.342 (3.096, 3.589) | 93.100 | Reference |
| Reeducation Centers (67) | 0.224 (0.157, 0.291) | 0.100 | 0.314 | 10.963 (9.761, 12.166) | 88.800 | **0.000** |
| Others (15) ^*^ | 0.324 (0.091, 0.558) | 82.300 | 1.000 | 4.620 (3.369, 5.871) | 94.700 | 1.000 |
| **Geographical location** | | | | | | |
| East China (104) | 0.029 (0.016, 0.042) | 0.000 | Reference | 5.616 (5.109, 6.122) | 95.500 | Reference |
| South China (76) | 0.238 (0.185, 0.291) | 0.000 | **0.001** | 6.625 (5.832, 7.418) | 94.500 | 0.533 |
| Southwest (39) | 2.287(1.774, 2.799) | 94.000 | **0.000** | 4.863 (3.969, 5.757) | 95.300 | 1.000 |
| Central China (25) | 0.121 (0.048, 0.194) | 0.000 | 0.914 | 2.461 (1.895, 3.026) | 68.800 | 0.370 |
| Northeast (18) | 0.123 (0.040, 0.206) | 0.000 | 0.809 | 1.025 (0.632, 1.418) | 73.400 | 0.111 |
| Northwest (25) | 0.137 (0.059, 0.215) | 0.000 | 0.680 | 2.788 (2.019, 3.558) | 91.300 | 0.126 |
| North China (54) | 0.056 (0.028, 0.084) | 0.000 | 0.967 | 3.237 (2.740, 3.734) | 0.900 | **0.011** |
| **Study design** | | | | | | |
| Cross-sectional studies (332) | 0.186(0.158, 0.214) | 57.200 | Reference | 4.389 (4.130, 4.649) | 94.000 | Reference |
| Others (9) ^†^ | 1.419 (0.481, 2.358) | 95.600 | 1.000 | 7.357 (4.398, 10.317) | 95.300 | 0.899 |
| **Sampling methods** | | | | | | |
| Multistage sampling (58) | 0.097 (0.061, 0.133) | 13.600 | Reference | 3.246 (2.783, 3.708) | 90.100 | Reference |
| Convenience sampling (278) | 0.224 (0.187, 0.261) | 69.100 | 1.000 | 4.766 (4.463, 5.068) | 89.900 | 1.000 |
| RDS/snowball sampling (5) ^‡^ | 0.203^§^ (-0.073, 0.479) | 45.100 | 0.224 | 6.196 (3.023, 9.369) | 94.600 | 0.999 |
| **Study time** | | | | | | |
| 2000-2002 (21) | 0.284 (0.080, 0.488) | 68.100 | Reference | 9.669 (7.81, 11.529) | 86.800 | Reference |
| 2003-2005 (80) | 0.211 (0.149, 0.273) | 30.000 | 0.999 | 4.970 (4.384, 5.556) | 94.900 | 1.000 |
| 2006-2008 (168) | 0.242 (0.190, 0.294) | 75.800 | 1.000 | 4.404 (4.032, 4.775) | 93.900 | 1.000 |
| 2009-2011 (72) | 0.093 (0.062, 0.125) | 17.900 | 1.000 | 3.169 (2.738, 3.600) | 92.400 | 1.000 |
| **HIV testing methods^¶^** | | | | | | |
| Confirmatory test (279) | 0.194 (0.163, 0.226) | 66.500 | Reference | — | — | — |
| Single method (24) | 0.151 (0.061, 0.241) | 0.000 | 1.000 | — | — | — |
| Unspecified (38) | 0.375 (0.202, 0.548) | 66.800 | 1.000 | — | — | — |
| **Syphilis testing methods^**^** | | | | | | |
| Treponemal tests (165) | — | — | — | 6.195 (5.728, 6.663) | 94.800 | Reference |
| Nontreponemal tests (148) | — | — | — | 2.977 (2.669, 3.284) | 93.100 | 0.300 |
| Unspecified (28) | — | — | — | 3.975 (3.039, 4.912) | 88.100 | 0.822 |
| **Overall** (341) | 0.203 (0.172, 0.233) | 65.000 | — | 4.475 (4.215, 4.735) | 94.100 | — |

^*^Comprising “Unspecified (6), Entertainment Venues and Reeducation Centers (6), Medical Institutions (1), Reeducation Centers and STD Clinics (1) and STD Clinics (1)”.

^†^Comprising “Cohort Study (1) and Intervention Study (8)”.

^‡^RDS: Respondent Driven Sampling.

^§^*p*> 0.05.

^||^Adjusted by the Monte Carlo permutation test with the number of random permutations being 10 000.

^¶^Sorted into three groups: Confirmatory tests (ELISA + Western blot, ELISA-1 + ELISA-2, ELISA-1 + ELISA-2 + Western blot, Immunocolloidal gold + Western blot, ELISA + Immunocolloidal gold + Western blot); Single method (single ELISA); and Unspecified (exact methods not presented although diagnosis of HIV infection based on positive serological tests).

^**^Sorted into three groups: Treponemal tests (*Treponema pallidum* particle agglutination assay – TPPA, Enzyme-Linked Immuno Sorbent Assay – ELISA, *Treponema pallidum* hemagglutination assay – TPHA, Immunocolloidal gold); Nontreponemal tests (Rapid plasma reagin – RPR, Toluidine red unheated serum test – TRUST, Unheated serum reagin – USR), and Unspecified (exact methods not presented although diagnosis of syphilis infection based on positive serological tests).
