# Supplementary material for: A Decline in HIV and Syphilis Epidemics in Chinese Female Sex Workers (2000–2011): A Systematic Review and Meta-Analysis
Source: PLoS One. 2013 Dec 13;8(12):e82451. doi: 10.1371/journal.pone.0082451 (PMC3862622; doi:10.1371/journal.pone.0082451)
Supplement: Table S6 — HIV and syphilis epidemic trends by subgroup in each three-year study period. (DOC) [file pone.0082451.s006.doc]

**Table S6. HIV and syphilis epidemic trends by subgroup in each three-year study period.**

|  | **2000–2002** | | **2003–2005** | | **2006–2008** | | **2009–2011** | |
| --- | --- | --- | --- | --- | --- | --- | --- | --- |
| **Subgroup** | **HIV Prevalence % (95% CI)** | **Syphilis Prevalence % (95% CI)** | **HIV Prevalence % (95% CI)** | **Syphilis Prevalence % (95% CI)** | **HIV Prevalence % (95% CI)** | **Syphilis Prevalence % (95% CI)** | **HIV Prevalence % (95% CI)** | **Syphilis Prevalence % (95% CI)** |
| **Language** | | | | | | | | |
| Chinese | 0.152 (0.054, 0.249) | 9.707 (7.740, 11.674) | 0.201 (0.141, 0.261) | 4.754 (4.176, 5.332) | 0.197 (0.152, 0.241) | 4.177 (3.811, 4.542) | 0.096 (0.064, 0.128) | 3.200 (2.760, 3.640) |
| English | 10.300 (7.649, 12.951) | 9.500 (6.943, 12.057) | 0.966 (0.241, 1.690) | 13.234 (8.633, 17.834) | 3.005 (1.615, 4.395) | 7.901 (5.825, 9.977) | 0.119 (-0.114, 0.351)|| | 2.293 (1.283, 3.304) |
| **Sample size** | | | | | | | | |
| <200 | 0.370 (0.034, 0.706) | 8.464 (5.339, 11.590) | 0.346 (0.053, 0.638) | 10.809 (6.913, 14.706) | 0.426 (0.235, 0.618) | 5.812 (4.414, 7.211) | 0.429 (0.075, 0.783) | 3.905 (1.821, 5.989) |
| 200–400 | 0.208 (0.015, 0.402) | 10.780 (7.097, 14.464) | 0.211 (0.144, 0.279) | 4.828 (4.118, 5.537) | 0.219 (0.149, 0.288) | 5.076 (4.431, 5.721) | 0.190 (0.111, 0.269) | 3.263 (2.572, 3.954) |
| ≥400 | 0.392 (-0.068, 0.852)|| | 9.864 (6.634, 13.094) | 0.205 (0.065, 0.345) | 3.957 (2.846, 5.068) | 0.229 (0.154, 0.304) | 3.687 (3.196, 4.179) | 0.081 (0.044, 0.119) | 3.133 (2.511, 3.754) |
| **Study location** | | | | | | | | |
| Entertainment Venues | 0.161 (-0.087, 0.410)|| | 11.256 (1.430, 21.082) | 0.171 (0.113, 0.229) | 2.923 (2.423, 3.423) | 0.235 (0.178, 0.291) | 3.437 (3.084, 3.789) | 0.100 (0.066, 0.135) | 3.239 (2.788, 3.689) |
| Reeducation Centers | 0.150 (0.044, 0.255) | 9.303 (7.513, 11.094) | 0.324 (0.135, 0.512) | 11.484 (9.123, 13.845) | 0.311 (0.176, 0.446) | 11.614 (9.587, 13.641) | 0.206 (-0.244, 0.655)|| | 14.237 (-7.022, 35.497)|| |
| Others^*^ | 10.300 (7.649, 12.951) | 9.500 (6.943, 12.057) | 0.994 (-0.134, 2.122)|| | 8.365 (1.667, 15.064) | 0.179 (-0.016, 0.375)|| | 4.879 (2.447, 7.311) | 0.095 (-0.036, 0.225)|| | 1.238 (0.122, 2.353) |
| **Geographical location** | | | | | | | | |
| East China | 0.200 (0.007, 0.393) | 12.052 (7.495, 16.608) | 0.075 (0.026, 0.124) | 5.460 (4.375, 6.546) | 0.024 (0.004, 0.044) | 6.763 (5.807, 7.719) | 0.026 (0.008, 0.044) | 3.617 (2.906, 4.328) |
| South China | 0.144 (-0.115, 0.403)|| | 15.631 (6.480, 24.782) | 0.228 (0.095, 0.361) | 11.391 (8.239, 14.542) | 0.240 (0.163, 0.317) | 5.705 (4.717, 6.692) | 0.254 (0.159, 0.348) | 3.803 (2.591, 5.016) |
| Southwest | 10.300 (7.649, 12.951) | 9.500 (6.943, 12.057) | 1.551 (0.502, 2.601) | 8.197 (4.567, 11.827) | 3.220 (2.402, 4.038) | 4.903 (3.663, 6.144) | 0.389 (0.184, 0.594) | 2.206 (1.174, 3.237) |
| Central China | - | - | 0.088 (-0.031, 0.207)|| | 2.014 (0.976, 3.052) | 0.170 (0.015, 0.325) | 3.210 (2.081, 4.339) | 0.125 (0.010, 0.240) | 2.147 (1.339, 2.955) |
| Northeast | 0.309 (-0.546, 1.163)|| | 9.300 (4.828, 13.772) | 0.134 (-0.053, 0.321)|| | 0.249 (-0.005, 0.503)|| | 0.119 (0.021, 0.216) | 1.117 (0.658, 1.576) | 0.112 (-0.198, 0.422)|| | 2.500 (1.053, 3.947) |
| Northwest | 0.439 (0.060, 0.817) | 5.692 (4.368, 7.015) | 0.265 (0.077, 0.453) | 1.945 (0.931, 2.959) | 0.196 (0.012, 0.379) | 1.555 (0.439, 2.670) | 0.063 (-0.038, 0.163)|| | 3.441 (-0.054, 6.936)|| |
| North China | 0.091 (-0.043, 0.224)|| | 7.039 (5.903, 8.176) | 0.166 (0.043, 0.289) | 3.209 (2.011, 4.406) | 0.043 (0.013, 0.073) ||240(0.163,0.317)00000000000000000000000000000000000000000000000000000000000000000000000000000000000000000000000000000000000000 | 2.913 (2.315, 3.511) | 0.164 (0.010, 0.317) | 2.708 (1.583, 3.833) |
| **Study design** | | | | | | | | |
| Cross-sectional studies | 0.306 (0.085, 0.527) | 9.816 (7.842, 11.791) | 0.211 (0.149, 0.273) | 4.970 (4.384, 5.556) | 0.210 (0.163, 0.257) | 4.257 (3.889, 4.625) | 0.041 (0.024, 0.058) | 3.126 (2.695, 3.558) |
| Others^†^ | 0.118 (-0.210, 0.447)|| | 7.580 (5.055, 10.105) | - | - | 2.069 (0.518, 3.619) | 7.541 (3.763, 11.318) | 0.401 (-0.189, 0.990)|| | 7.226 (-5.313, 19.765)|| |
| **Sampling methods** | | | | | | | | |
| Multistage sampling | 0.276 (-0.488, 1.041)|| | 3.310 (0.704, 5.916) | 0.255 (0.021, 0.490) | 5.084 (1.982, 8.186) | 0.062 (.025, 0.098) | 3.574 (2.901, 4.248) | 0.136 (0.060, 0.211) | 2.659 (2.010, 3.309) |
| Convenience sampling | 0.287 (0.076, 0.498) | 10.006 (8.124, 11.889) | 0.202 (0.139, 0.264) | 4.898 (4.294, 5.501) | 0.284 (0.218, 0.351) | 4.632 (4.191, 5.073) | 0.044 (0.22, 0.066) | 3.454 (2.897, 4.011) |
| RDS^¶^/snowball sampling | - | - | 2.300 (0.738, 3.862) | 11.000 (7.741, 14.259) | 0.149 (-0.090, 0.388)|| | 6.167 (2.221, 10.113) | 0.116 (-0.205, 0.436)|| | 2.200 (0.817, 3.583) |
| **HIV testing methods**^‡^ | | | | | | | | |
| Confirmatory test | 0.308 (0.075, 0.541) | - | 0.209 (0.141, 0.277) | - | 0.226 (0.172, 0.280) | - | 0.096 (0.061, 0.131) | - |
| Single method | 0.595 (-1.050, 2.240)|| | - | 0.233 (0.047, 0.419) | - | 0.219 (0.022, 0.416) | - | 0.089 (-0.031, 0.209) | - |
| Unspecified | 0.143 (-0.159, 0.445)|| | - | 0.638 (-0.064, 1.339)|| | - | 0.539 (0.225, 0.852) | - | 0.175 (0.027, 0.324) | - |
| **Syphilis testing methods**^§^ | | | | | | | | |
| Treponemal tests | - | 9.455 (7.664, 11.246) | - | 8.734 (7.045, 10.422) | - | 6.291 (5.584, 6.998) | - | 3.802 (3.131, 4.473) |
| Nontreponemal tests | - | 15.187 (0.560, 30.935) | - | 3.214 (2.630, 3.799) | - | 2.754 (2.344, 3.163) | - | 2.549 (1.935, 3.163) |
| Unspecified | - | 5.458 (1.273, 9.642) | - | 4.569 (1.302, 7.835) | - | 4.763 (3.230, 6.296) | - | 2.212 (1.017, 3.407) |
| **Overall** | 0.284 (0.080, 0.488) | 9.669 (7.810, 11.529) | 0.211 (0.149, 0.273) | 4.970 (4.384, 5.556) | 0.242 (0.190, 0.294) | 4.404 (4.032, 4.775) | 0.041 (0.024, 0.058) | 3.169 (2.738, 3.600) |

A random effect model (*Q* test *p* < 0.10) or a fixed effect model was used (*Q* test *p* > 0.10).

^*^Comprising “Unspecified (6), Entertainment Venues and Reeducation Centers (6), Medical Institutions (1), Reeducation Centers and STD Clinics (1) and STD Clinics (1)”.

^†^Comprising “Cohort Study (1) and Intervention Study (8)”.

^‡^Sorted into three groups: Confirmatory tests (ELISA + Western blot, ELISA-1 + ELISA-2, ELISA-1 + ELISA-2 + Western blot, Immunocolloidal gold + Western blot, ELISA + Immunocolloidal gold + Western blot); Single method (single ELISA); and Unspecified (exact methods not presented although diagnosis of HIV infection based on positive serological tests).

^§^Sorted into three groups: Treponemal tests (*Treponema pallidum* particle agglutination assay-TPPA, Enzyme-Linked Immuno Sorbent Assay-ELISA, *Treponema pallidum* hemagglutination assay-TPHA, Immunocolloidal gold); Nontreponemal tests (Rapid plasma reagin-RPR, Toluidine red unheated serum test-TRUST, Unheated serum reagin-USR); and Unspecified (exact methods not presented although diagnosis of syphilis infection based on positive serological tests).

^||^*p* > 0.05

^¶^RDS: Respondent Driven Sampling.
