# Supplementary material for: A Decline in HIV and Syphilis Epidemics in Chinese Female Sex Workers (2000–2011): A Systematic Review and Meta-Analysis
Source: PLoS One. 2013 Dec 13;8(12):e82451. doi: 10.1371/journal.pone.0082451 (PMC3862622; doi:10.1371/journal.pone.0082451)
Supplement: Table S7 — Distribution of HIV/syphilis prevalence datasets and sample sizes between subgroups and the four three-year study periods. (DOC) [file pone.0082451.s007.doc]

**Table S7. Distribution of HIV/syphilis prevalence datasets and sample sizes between subgroups and the four three-year study periods.**

|  | **2000–2002** | | **2003–2005** | | **2006–2008** | | **2009–2011** | |
| --- | --- | --- | --- | --- | --- | --- | --- | --- |
| **Subgroup** | **HIV dataset (total sample size)** | **Syphilis dataset (total sample size)** | **HIV dataset (total sample size)** | **Syphilis dataset (total sample size)** | **HIV dataset (total sample size)** | **Syphilis dataset (total sample size)** | **HIV dataset (total sample size)** | **Syphilis dataset (total sample size)** |
| **Language** | | | | | | | | |
| Chinese | 20 (6155) | 20 (6155) | 78 (30096) | 78 (30096) | 160 (72372) | 160 (72153) | 70 (53017) | 70 (52905) |
| English | 1 (505) | 1 (505) | 2 (697) | 2 (697) | 8 (4460) | 8 (4460) | 2 (843) | 2 (843) |
| **Sample size** | | | | | | | | |
| <200 | 8 (1254) | 8 (1254) | 11 (1548) | 11 (1548) | 35 (4412) | 35 (4412) | 9 (1309) | 9 (1311) |
| 200–400 | 7 (2130) | 7 (2130) | 54 (17617) | 54 (17617) | 75 (24237) | 75 (24235) | 33 (11567) | 33 (11638) |
| ≥400 | 6 (3276) | 6 (3276) | 15 (11628) | 15 (11628) | 58 (48183) | 58 (47966) | 30 (40984) | 30 (40799) |
| **Study location** | | | | | | | | |
| Entertainment Venues | 3 (1006) | 3 (1006) | 55 (19484) | 55 (19484) | 135 (68528) | 135 (68309) | 66 (51336) | 66 (51224) |
| Reeducation Centers | 17 (5149) | 17 (5149) | 21 (6933) | 21 (6933) | 27 (6501) | 27 (6501) | 2 (390) | 2 (390) |
| Others^*^ | 1 (505) | 1 (505) | 4 (4376) | 4 (4376) | 6 (1803) | 6 (1803) | 4 (2134) | 4 (2134) |
| **Geographical location** | | | | | | | | |
| East China | 8 (2054) | 8 (2054) | 26 (11991) | 26 (11991) | 42 (23420) | 42 (23203) | 28 (30287) | 28 (30284) |
| South China | 3 (823) | 3 (823) | 17 (4932) | 17 (4932) | 44 (15462) | 44 (15462) | 12 (10898) | 12 (10716) |
| Southwest | 1 (505) | 1 (505) | 6 (2948) | 6 (2948) | 23 (10217) | 23 (10215) | 9 (3535) | 9 (3606) |
| Central China | 0 (0) | 0 (0) | 6 (2387) | 6 (2387) | 10 (2698) | 10 (2698) | 9 (3638) | 9 (3640) |
| Northeast | 1 (162) | 1 (162) | 4 (1474) | 4 (1474) | 12 (4788) | 12 (4788) | 1 (447) | 1 (447) |
| Northwest | 5 (1170) | 5 (1170) | 9 (2856) | 9 (2856) | 7 (2216) | 7 (2216) | 4 (2392) | 4 (2392) |
| North China | 3 (1946) | 3 (1946) | 12 (4205) | 12 (4205) | 30 (18031) | 30 (18031) | 9 (2663) | 9 (2663) |
| **Study design** | | | | | | | | |
| Cross-sectional studies | 20 (6238) | 20 (6238) | 80 (30793) | 80 (30793) | 162 (73318) | 162 (73099) | 70 (53420) | 70 (53308) |
| Others^†^ | 1 (422) | 1 (422) | 0 (0) | 0 (0) | 6 (3514) | 6 (3514) | 2 (440) | 2 (440) |
| **Sampling methods** | | | | | | | | |
| Multistage sampling | 1 (181) | 1 (181) | 5 (1776) | 5 (1776) | 32 (17741) | 32 (17741) | 20 (19379) | 20 (19197) |
| Convenience sampling | 20 (6479) | 20 (6479) | 74 (28663) | 74 (28663) | 133 (58090) | 133 (57871) | 51 (34049) | 51 (34119) |
| RDS^‡^/snowball sampling | 0 (0) | 0 (0) | 1 (354) | 1 (354) | 3 (1001) | 3 (1001) | 1 (432) | 1 (432) |
| **HIV testing methods^§^** | | | | | | | | |
| Confirmatory test | 18 (5973) | - | 66 (26960) | - | 139 (68317) | - | 56 (48455) | - |
| Single method | 1 (84) | - | 10 (2591) | - | 8 (2158) | - | 5 (2365) | - |
| Unspecified | 2 (603) | - | 4 (1242) | - | 21 (6357) | - | 11 (3040) | - |
| **Syphilis testing methods||** | | | | | | | | |
| Treponemal tests | - | 17 (5308) | - | 31 (10217) | - | 76 (39797) | - | 41 (28436) |
| Nontreponemal tests | - | 2 (749) | - | 45 (19173) | - | 78 (32341) | - | 23 (22922) |
| Unspecified | - | 2 (603) | - | 4 (1403) | - | 14 (4475) | - | 8 (2390) |
| **Overall** | 21 (6660) | 21 (6660) | 80 (30793) | 80 (30793) | 168 (76832) | 168 (76613) | 72 (53860) | 72 (53748) |

^*^Comprising “Unspecified (6), Entertainment Venues and Reeducation Centers (6), Medical Institutions (1), Reeducation Centers and STD Clinics (1) and STD Clinics (1)”.

^†^Comprising “Cohort Study (1) and Intervention Study (8)”.

^‡^RDS: Respondent Driven Sampling.

^§^Sorted into three groups: Confirmatory tests (ELISA + Western blot, ELISA-1 + ELISA-2, ELISA-1 + ELISA-2 + Western blot, Immunocolloidal gold + Western blot, ELISA + Immunocolloidal gold + Western blot); Single method (single ELISA); and Unspecified (exact methods not presented although diagnosis of HIV infection based on positive serological tests).

^||^Sorted into three groups: Treponemal tests (*Treponema pallidum* particle agglutination assay – TPPA, Enzyme-Linked Immuno Sorbent Assay – ELISA, *Treponema pallidum* hemagglutination assay – TPHA, Immunocolloidal gold); Nontreponemal tests (Rapid plasma reagin – RPR, Toluidine red unheated serum test – TRUST, Unheated serum reagin – USR); and Unspecified (exact methods not presented although diagnosis of syphilis infection based on positive serological tests).
